# Synthesis of New Steroidal Carbamates with Plant-Growth-Promoting Activity: Theoretical and Experimental Evidence

**DOI:** 10.3390/ijms22052330

**Published:** 2021-02-26

**Authors:** Daylin Fernández Pacheco, Leonardo González Ceballos, Armando Zaldo Castro, Marcos R. Conde González, Laura González de la Torre, Lia Pérez Rostgaard, Luis Espinoza, Katy Díaz, Andrés F. Olea, Yamilet Coll García

**Affiliations:** 1 Center for Natural Product Research, Faculty of Chemistry, University of Havana, Zapata and G, Havana 10400, Cuba; daylinfp@fq.uh.cu (D.F.P.); leo@fq.uh.cu (L.G.C.); zaldo@fq.uh.cu (A.Z.C.); mrconde@fq.uh.cu (M.R.C.G.); lrostgaard@estudiantes.fq.uh.cu (L.P.R.); 2 Sierra Maestra Science, Technology and Innovation Entity, Barlovento Complex, 5ta Ave. and 246, Havana 11300, Cuba; ltorre@bionaturasm.cu; 3 Departamento de Química, Universidad Técnica Federico Santa María, Avenida España 1680, Valparaíso 2340000, Chile; katy.diaz@usm.cl; 4 Instituto de Ciencias Químicas Aplicadas, Facultad de Ingeniería, Universidad Autónoma de Chile, El Llano Subercaseaux 2801, Santiago 8900000, Chile; andres.olea@uautonoma.cl

**Keywords:** brassinosteroid analogs, plant-growth promoters, molecular docking, carbamates, rice lamina inclination test

## Abstract

A priority of modern agriculture is to use novel and environmentally friendly plant-growth promoter compounds to increase crop yields and avoid the indiscriminate use of synthetic fertilizers. Brassinosteroids are directly involved in the growth and development of plants and are considered attractive candidates to solve this problem. Obtaining these metabolites from their natural sources is expensive and cumbersome since they occur in extremely low concentrations in plants. For this reason, much effort has been dedicated in the last decades to synthesize brassinosteroids analogs. In this manuscript, we present the synthesis and characterization of seven steroidal carbamates starting from stigmasterol, β-sitosterol, diosgenin and several oxygenated derivatives of it. The synthesis route for functionalization of diosgenin included epoxidation and epoxy opening reactions, reduction of carbonyl groups, selective oxidation of hydroxyl groups, among others. All the obtained compounds were characterized by ^1^H and ^13^C NMR, HRMS, and their melting points are also reported. Rice lamina inclination test performed at different concentrations established that all reported steroidal carbamates show plant-growth-promoting activity. A molecular docking study evaluated the affinity of the synthesized compounds towards the BRI1-BAK1 receptor from *Arabidopsis thaliana* and three of the docked compounds displayed a binding energy lower than brassinolide.

## 1. Introduction

**Figure 1:**
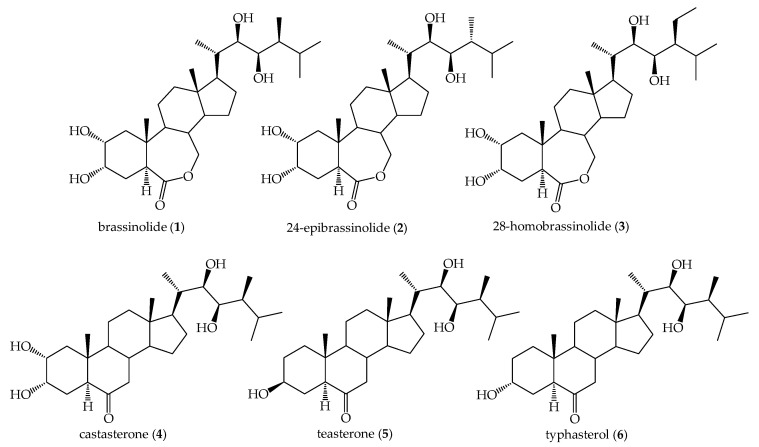
Representative natural occurring brassinosteroids.

The increase of crop yields is a common goal for all research groups dedicated to supplying agriculture with novel plant-growth enhancers. In this effort, the quest for biologically active compounds that are also friendly to the environment is of great interest. The growth and development of plants are regulated by endogenous factors (i.e., phytohormones) and exogenous factors (i.e., plant growth regulators). Within the first group, hormones and plant growth regulators play an indispensable role in the regulation of developmental processes. It is known that brassinosteroids (BRs), a family of phytohormones of steroidal nature [1,2], are involved in promoting cell expansion, cell elongation, cell division, and vascular differentiation [3,4,5,6,7,8], additionally BRs provides protection against environmental stress [9,10,11]. Some representative natural occurring BRs are shown in Figure 1.

Since the discovery of BRs in the seventies [1], much attention has been paid to these molecules because of their recognized participation in physiological processes beginning in embryonic development up to homeostasis in the adult plant [12]. In this way they become very promising compounds to be used to increase agricultural yields. Besides their role as enhancers of agriculture production yields [13,14], BRs are considered environmentally safe for providing plant protection [10]. However, the very low concentration of BRs in plants is the main drawback for their extensive use [4]. Therefore, for practical purposes, chemical synthesis appears as “the only” source of BRs and/or its analogs. Synthetic advances have allowed obtaining a series of BRs analogs with novel structures and substituent functions, which exhibit similar or even higher biological effects as compared to natural molecules [15,16]. Thus, in the last years different research groups have reported the synthesis of new BRs analogs to study their structure/activity relationship [16,17,18,19,20], resulting in very active and stable compounds [21,22]. Several studies have proved that some synthetic BRs analogs can produce a wide range of physiological responses in plants, like potentiate plant growth and development and protection from several conditions of stress [15,23,24]. Otherwise, different nitrogen-containing compounds such as polyamines [25], acyl thioureas [26,27], thiazoles, oxadiazoles, and pyrimidines exhibit a broad biological activity spectrum and their potential properties as plant growth promoters are of great interest [28]. Besides, the synthesis of new aryl-brassinosteroid derivatives and their evaluation as plant growth promoters have been recently reported [29,30].

This work presents the synthesis of a series of BRs carbamates analogs. Various hydroxysteroids (stigmasterol, β-sitosterol, diosgenin, and oxygenated derivatives) were condensed with phenyl isocyanate to afford the corresponding carbamates [31]. Post condensation reactions, i.e., epoxidation, carbonyl reduction, and oxime formation were used to introduce new diversification elements. All the obtained compounds were characterized by NMR (^1^H and ^13^C) and HRMS. The growth-promoting activity of these BRs analogs was determined by using the rice-lamina inclination test due to its simplicity and selectivity [32]. A molecular docking study, using BRs receptor from *Arabidopsis thaliana* (BRI1-BAK1) as the model receptor and the final carbamates as ligands, was performed to provide theoretical insights into the experimental findings.

## 2. Results and Discussion

### 2.1. Synthesis

Diosgenin (**6**) has been used to prepare a significant number of spirostanic BRs analogs, which have shown a promising plant growth-promoting activity [17,33,34,35,36,37]. Considering these antecedents, we were interested in the possibility of preparing new diosgenin derivatives with the incorporation of carbamate functions in C3, modifications to ring B and evaluating the effect that these chemical modifications could have on plant growth-promoting activity.

**Scheme 1:**
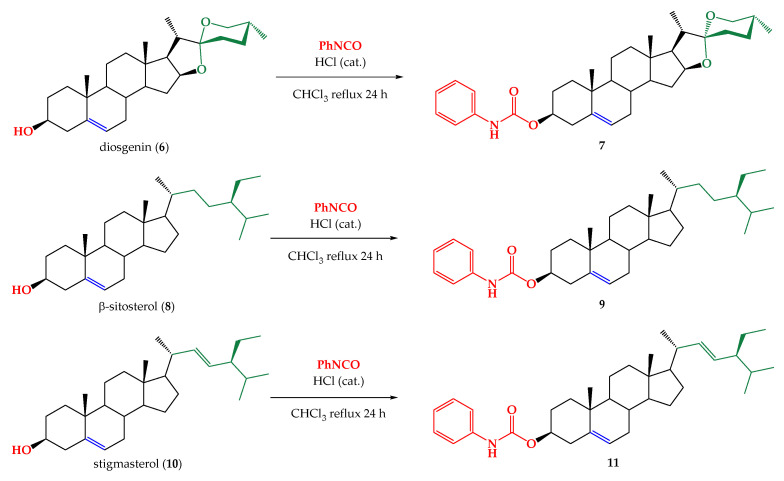
Synthesis of diosgenin, β-sitosterol and stigmasterol carbamates **7**, **9** and **11.**

The synthesis of corresponding carbamates of diosgenin, β-sitosterol and stigmasterol was carried out by reacting these starting compounds with phenyl isocyanate, using CHCl_3_ as solvent and HCl as acid catalyst (Scheme 1) [38,39,40].

The reactions were monitored by thin-layer chromatography (TLC) for 24 h. After completion, the crudes were purified by column chromatography to afford the respective carbamates. The reaction yields were 62%, 73% and 71% for compounds **7**, **9** and **11**, respectively. The main spectroscopic evidence for obtaining of these products comes from NMR spectroscopy. In the ^1^H NMR spectra aromatic H give signals at *δ*_H_ = 7.40–7.04 ppm and H-N produces a signal between *δ*_H_ = 6.58-6.53 ppm, for the three compounds. On the other hand, in the ^13^C NMR spectra, the signals observed at *δ*_C_ = 153.16, 153.24 and 153.26 ppm were assigned to the carbamoyl group (NHCO) of compounds **7**, **9** and **11**, respectively.

**Scheme 2:**
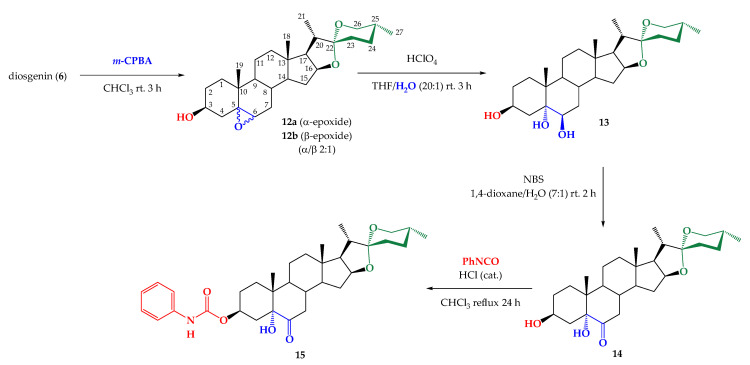
Synthesis of a B-ring functionalized derivative of diosgenin carbamate.

Synthesis of carbamate **15** from diosgenin goes through the intermediate **14** that is obtained by a three-step sequence including epoxidation of diosgenin with *meta*-chloroperoxybenzoic acid (*m*-CPBA) [41], acid-catalyzed epoxide opening [42], and selective oxidation of axial -OH group at position C6 with *N*-bromosuccinimide (NBS) [43] (Scheme 2).

**Figure 2:**
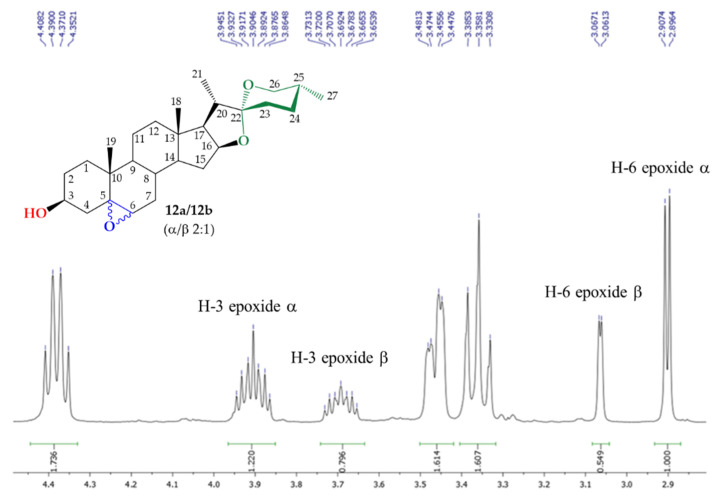
^1^H NMR spectrum (expansion zone 2.80-4.50 ppm) of diasteroisomeric epoxides mixture **12a**/**12b.**

Epoxidation of diosgenin (**6**) originates a mixture of α- and β-epoxides with 95% yield. The diastereomeric ratio of each epoxide in the mixture was established by integration of ^1^H NMR signals assigned to the H-3 epoxide α (compound **12a**) which in the mixture appear at *δ*_H_ = 3.95-3.86 ppm (multiplet) and H-3 epoxide β (compound **12b**) which appear at *δ*_H_ = 3.73-3.65 ppm (multiplet), and H-6 epoxide β (compound **12b**) at *δ*_H_ = 3.06 ppm (d, *J* = 2.3 Hz) and H-6 epoxide α (compound **12a**) at *δ*_H_ = 2.90 ppm (d, *J* = 4.4 Hz) (Figure 2). Based on these NMR measurements the relative ratio of **12a**:**12b** was determined as 2:1. Additionally, it was possible to assign most of the ^1^H and ^13^C NMR signals from the **12a**/**12b** epoxide mixture.

Opening of the epoxides mixture, in acid medium (HClO_4_/THF/H_2_O), generates just one diastereomer (**13**, 78% yield) due to steric hindrance induced by the methyl group at position C19 [42]. Compound **14** was obtained from **13** by stereo and regio-selective oxidation with NBS (83% yield) [43]. Obtention of **14** was confirmed by ^13^C NMR, i.e., signal at *δ*_C_ = 212.09 ppm assigned to the carbonyl group at C6. Treatment of ketone **14** with phenyl isocyanate under acidic conditions produces carbamate derivative **15** in 68% yield. The presence of signals at *δ*_H_ = 7.32-7.00 and 6.60 ppm in the ^1^H NMR spectrum are assigned to hydrogen atoms from the aromatic ring and the carbamoyl group (NHCO), respectively, confirming the presence of carbamoyl group in compound **15**.

**Scheme 3:**
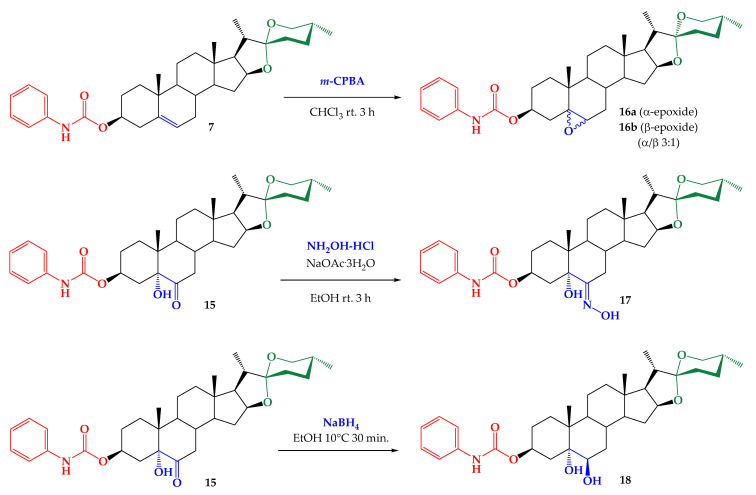
Derivatization of diosgenin carbamates, obtaining compound **16**–**18**.

Epoxidation of diosgenin carbamate (**7**) with *m*-CPBA produced the **16a**/**16b** mixture with 94% yield (Scheme 3).

**Figure 3:**
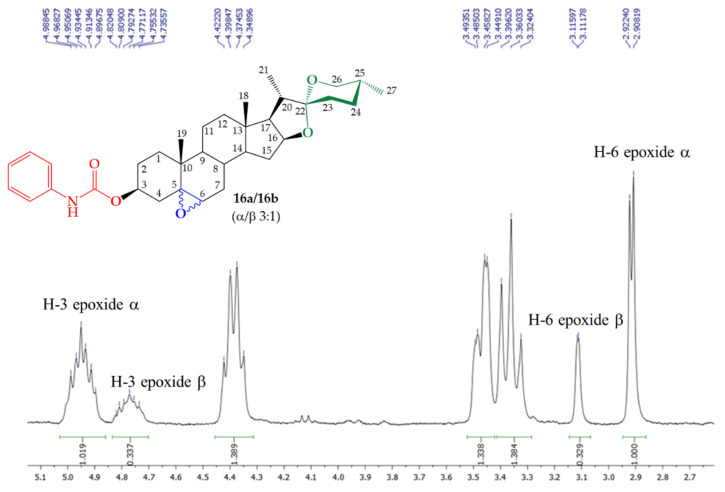
^1^H NMR spectrum (expansion zone 2.70-5.10 ppm) of diasteroisomeric epoxides mixture **16a**/**16b**.

The diastereomeric ratio of each epoxide in the mixture was established by NMR spectroscopy in the same way as described above for the mixture **12a**/**12b**. So, integration of ^1^H NMR signals assigned to the H-3 epoxide α (compound **16a**) which appear at *δ*_H_ = 4.99-4.90 ppm (multiplet) and H-3 epoxide β (compound **16b**) which appear at *δ*_H_ = 4.82-4.74 ppm (multiplet), and the signals at *δ*_H_ = 3.11 ppm (d, *J* = 1.7 Hz) and *δ*_H_ = 2.92 ppm (d, *J* = 5.7 Hz) assigned to the H-6β and H-6α, respectively (Figure 3). Based on these NMR measurements the relative ratio of **16a**:**16b** obtained was determined as 3:1.

On the other hand, treatment of carbamate 15 with NH_2_OH^.^HCl/NaOAc^.^3H_2_O system (according to reported protocols [44,45]), produces oxime 17 with 77% yield. Spectroscopic evidence of transformation of ketone group into hydroxy oxime function in C6 is the appearance of a singlet signal at *δ*_H_ = 10.51 ppm, assigned at N-OH, in the ^1^H NMR spectrum of compound 17. While in the ^13^C NMR spectrum of compound 17, the C6 signal was observed at *δ*_C_ = 159.16 ppm, compared to the chemical shift observed for C6 in precursor 15, which was observed at *δ*_C_ = 211.98 ppm. Finally, reduction of the ketone group (C6) in compound 15 with NaBH_4_/EtOH, occurs in a stereospecific way producing compound 18 with 83% yield. The stereospecificity of the reduction could be explained in terms of the steric hindrance offered by the CH_3_-19 attached to C10, which force the reducing agent to be added through the lower face of ring B. The main spectroscopic evidence that confirms the obtaining of derivative 18 was the observation of the signal at *δ*_H_ = 4.58 ppm (d, *J* = 4.2 Hz) assigned to the C6-OH group in the ^1^H NMR spectrum of compound 18. While in the ^13^C NMR spectrum of compound 18, the carbinolic C6 signal was observed at *δ*_C_ = 73.63 ppm, compared to the chemical shift observed for C6 in precursor 15, which was observed at *δ*_C_ = 211.98 ppm.

### 2.2. Rice-Lamina Inclination Test

The rice-lamina inclination test is a very simple and selective method that is widely used to measure activity of BRs analogs as plant-growth promoters [32]. In this assay, the angle formed between the laminae and sheaths of rice seedlings is determined, and significant differences observed in treated seedlings are associated to BRs-induced increase on plant growth. Thus, a higher angle degree indicates higher activity of applied BRs analogs. This study was conducted at three different concentrations and two bioassays with several replicas were carried out for statistical purposes. Results obtained are shown in Table 1. Brassinolide was taken as positive control since it is the most active natural BRs and distilled water was used as negative control.

All compounds induce angle degrees higher than the one measured for the negative control but lower than the respective value obtained for brassinolide. Nonetheless, a comparison of activities shown by compounds synthesized in this work evidences some interesting structure-activity results. At low concentrations, the activities of carbamates derived from stigmasterol and β-sitosterol are similar to that found for brassinolide (Activity ratio Analog/Brassinolide = 1 to 0.5), and higher than that measured for the diosgenin carbamate. However, this effect is lost at 1.0 µM. The main structural difference between these compounds lays in the side chain, i.e., the bulky glycosyl group increase the hydrophobicity and steric effects of any interaction involving this part of the molecule. On the other hand, at all tested concentrations, analogs **15**, **16a**/**16b**, **17** and **18** exhibit higher activities than that observed for diosgenin-derived carbamate **7**. These compounds differ from **7** just in the substitution in C5 and C6. Thus, it can be concluded that presence of oxygenated functionalities in positions C5 and C6 are key factors in determining growth-promoting activity of diosgenin carbamates. In this sense, an epoxide group linking C5 and C6 (mixture **16a**/**16b**) or carbonyl group in C6 (compound **15**) seems to be more effective than hydroxyl or hydroxymino groups in inducing activity. Interestingly, mixture **16a**/**16b** shows the higher activities of all analogs and at the intermediate concentration is as active as brassinolide. The advantage for a potential application lays in its synthesis which is relatively straightforward and with high yields.

### 2.3. Molecular Docking Study

Previously, a combination of genetic, biochemical, and proteomic approaches has accelerated the understanding of the BRs signaling pathway in *Arabidopsis thaliana* [46]. The signal transduction pathway of BR has been intensively studied the past decade [46,47,48] and these studies have established a complex BR signal transduction pathway, that play an important role in plant growth and development. Structural analysis revealed that a hydrophobic groove, formed between the inner surface of the helical BRI1-LRR and a ~70-residues island domain, is responsible for the specific recognition of **1** by BRI1 [49]. The binding site of BRI1 is located on the surface of the receptor ectodomain as a nonpolar cleft lined by nonpolar aromatic and aliphatic residues (I540, I563, W564, Y599, Y642, M657, F681, I682, I706), whereas hydroxyl groups form the cleft ridge (Y597, Y599, Y642, S647). Brassinolide fits into the cleft via its nonpolar side and displays its hydroxyl groups towards the solvent and protein partners [50]. At this point, **1** induces heterodimerization between BRI1 and the somatic embryogenesis receptor kinases (SERKs) family of co-receptors [51]. This leads to BRI1-SERK trans-phosphorylation and subsequent downstream signaling mediating plant growth and development.

Molecular docking is a valuable computational tool to predict the interaction geometry and strength between two macromolecules or, between a small ligand and a macromolecule, starting from their unbound conformations [52]. It is known that BRs perception occurs in a two-step mechanism [53]. First, BRs binds to a hydrophobic surface groove on receptor BRI1-LRR domain, and this is followed by the heterodimerization with BAK1/SERK1-LRR, fully activating the receptor. To gain further insight into the activity of the new steroidal carbamates introduced in this study, we performed rigid docking of the ligands into the active site of the BRI1-BAK1 complex (PDB: 4m7e) using AutoDock Vina [52] since crystal structures of BRI1 and BAK1 are available.

**Figure 4:**
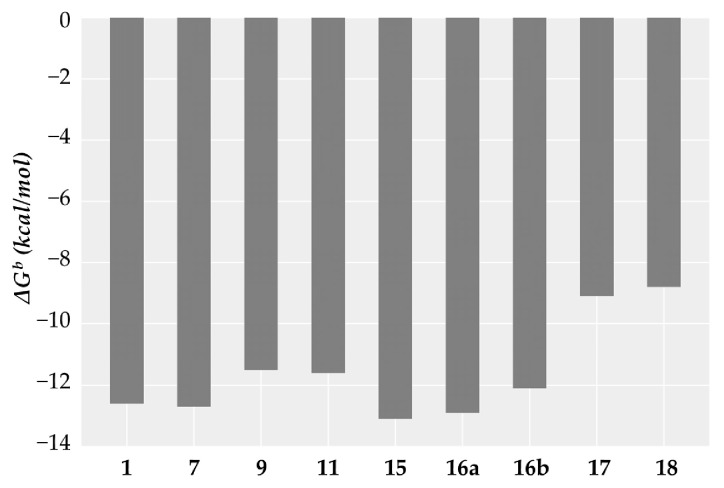
Binding energies between **1**, **7**, **9**, **11**, **15**, **16a**, **16b**, **17**, **18** and BRI1-BAK1 complex.

Redocking of the natural ligand, **1**, was performed to assess the quality of the search parameters. Results show that the lowest energy pose is almost identical to the crystallographic pose, so the parameters were considered appropriate and docking of synthetic ligands was carried out. The poses considered representative for each ligand align well with **1** in the binding site. All Vina binding energies for the complexes were negative (Figure 4) suggesting that formation of the BRI1–ligand–BAK1 complex is favored.

**Figure 5:**
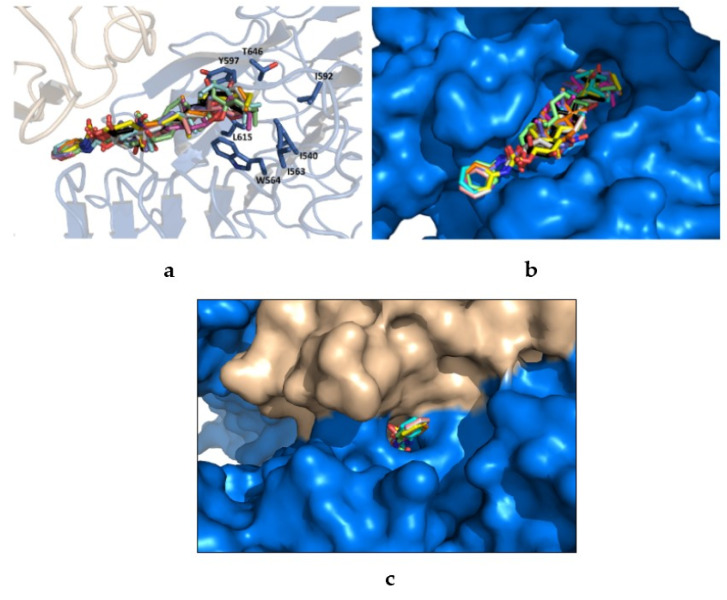
General picture of the ligand poses. (**a**) Hydrophobic side chains of all carbamates are buried into a hydrophobic pocket. (**b**) Active site with hidden BAK1 for clarity. (**c**) Phenyl moiety of all carbamates protrudes out of the binding site. BRI1 (blue), BAK1 (wheat), compounds **7**, **9**, **11**, **15**, **16a**, **16b**, **17** and **18** are depicted as color sticks, whereas brassinolide appears colored black.

As can be seen in Figure 5a, the hydrophobic side chains of all carbamates are buried in the hydrophobic pocket delineated by residues I540, I563, W564, I592, Y597, L615 and T646. However, since these carbamates are larger than **1**, they occupy a larger volume in the active site, and thus the phenyl groups slightly protrude out of the binding site (Figure 5b,c). Although it would seem reasonable that the phenyl group could also fit in the hydrophobic pocket, as has been reported for other analogs [50], the lack of functionalities that promote the interactions between both proteins and the carbamates in the inverted conformation, makes it unfavorable. This could be the reason for the modest activities determined for carbamates as compared to **1**.

**Figure 6:**
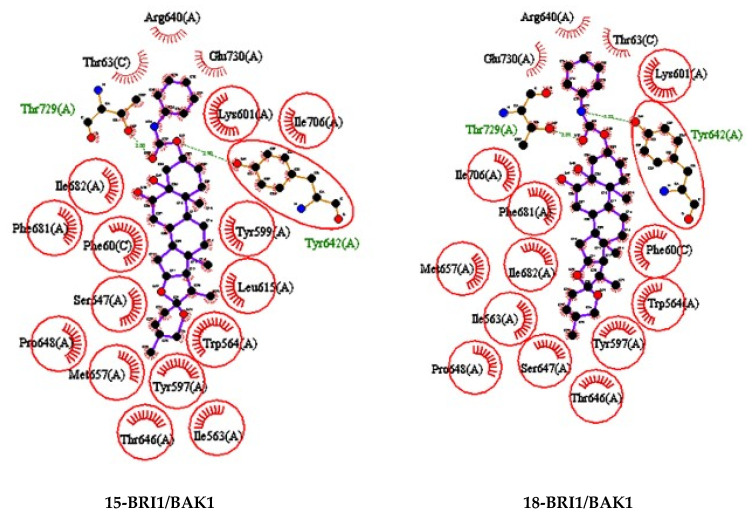
Ligand–Protein contacts for steroidal carbamates 15 and 18. Circled residues represent common interactions between 1 and synthetic ligands. For the other studied compounds see Figure S12.

To gain further insight into the activities of the compounds we also analyzed the ligand-proteins interactions with the software LigPlot+ (see Figure S12, Supplementary Materials, for all interaction diagrams) [54,55]. As can be seen in Figure 6, the interaction diagrams of steroidal carbamates **15** and **18** show the amino acid residues of the active site participating in the stabilization of complexes BRI1-ligand-BAK1. Circled residues represent common interactions between **1** and the complexes. As expected, the number of common interactions observed for carbamate **15** is bigger than that corresponding to **18**. This result is in line with the binding energy values obtained for both carbamates since **15** possesses the lowest binding energy and **18** the highest of all synthesized carbamates. Among all possible interactions between ligands and proteins, hydrophobic packing interactions are predominant. This is not surprising, considering the hydrophobic nature of the binding pocket formed by the interaction of BRI1 with BAK1. Some important hydrogen bonds (H41 and V62) are absent for all compounds [53], and this is other fact that could contribute to the reduced activity of carbamates as compared to **1**. On the other hand, there is a hydrogen bond interaction with Y642, which has proved to be important for binding affinity [54,55], and a new hydrogen bond between the carbonyl moiety of the phenyl carbamate and the side chain hydroxyl group of T729 [56]. These interactions are present in several compounds with different activity profiles, which underlines the complexity of BRs signaling in plants and the intrinsic inaccuracy of Vina scoring function [57]. Assuming that these poses are appropriate representations of the actual binding mode of steroidal carbamates **7**, **9**, **11**, **15**, **16a**, **16b**, **17**, and **18**, several conclusions regarding functional groups diversity can be inferred. For example, the presence of oxygenated functionalities on ring A besides the carbamoyl group can improve the interactions with the BAK1 cofactor, necessary for proper receptor activation. Moreover, these kinds of functionalities in the side chains could also contribute to these interactions.

**Figure 7:**
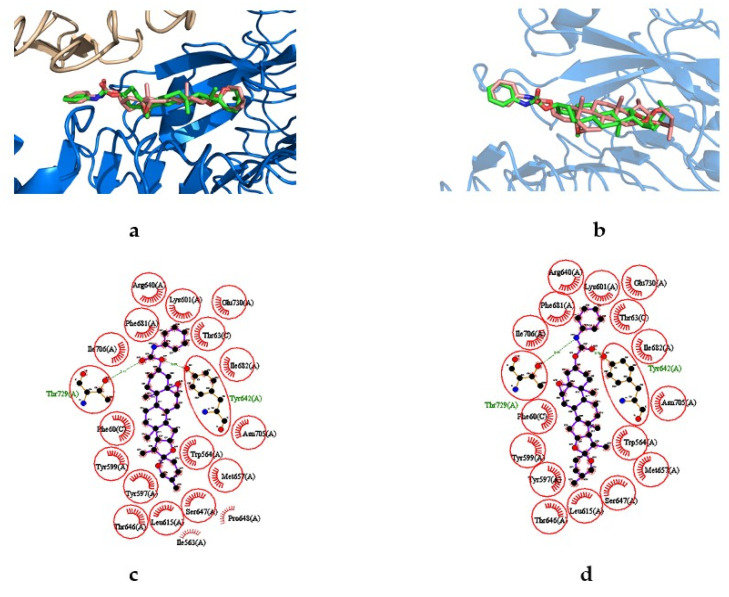
Binding modes and protein contacts of diastereomers (**a**) compound **16a** (green sticks), (**b**) compound **16b** (pink sticks), (**c**) Protein contacts of compound 16a. (**d**) Protein contacts of compound **16b**. Circled residues represent shared contacts. BRI1 (blue), BAK1 (wheat). For the other studied compounds see Figures S11 and S12

For compounds **16a** and **16b**, α and β epoxides respectively, the docking studies indicate that both diastereomers, although presenting two distinct binding modes to the BRI1/BAK1 heterodimer (Figure 7a,b), share almost the same protein contacts (Figure 7c,7d) and bind with approximately the same strength. Therefore, adaptation to the new geometry of the steroid due to the β-epoxide could be invoked to explain the change in binding mode while retaining the favorable contacts with the proteins.

## 3. Materials and Methods.

### 3.1. Chemical

#### General

All chemical reagents were purchased from Merck, Fluke, or Sigma-Aldrich and used without previous purification. All solvents were distilled and stored over proper desiccants. Melting points were measured on a BUCHI M-565 equipment (BÜCHI Labortechnik GmbH, Essen, Germany) being EtOAc the last solvent used in all cases. NMR spectra were recorded at 298 K on a Varian Mercury 400 NMR spectrometer (Varian, Palo Alto, CA, USA) at 400 MHz and 101 MHz for ^1^H and ^13^C respectively. All spectra were referenced using the TMS signal or the residual peak of the solvent. Chemical shifts (δ) are reported in ppm and coupling constants (*J*) are given in Hz. A TripleToF 6600-1 mass spectrometer (Sciex, MA, USA) was used for high-resolution mass spectrometry (HRMS). Silica gel (Merck 70–230 mesh) was used for column chromatography (CC) and silica gel plates HF254 for thin-layer chromatography (TLC). TLC spots were detected by heating after staining with cerium molybdate in H_2_SO_4_.

### 3.2. Methods of Synthesis

#### 3.2.1. Synthesis of Carbamates

To a solution of diosgenin, β-sitosterol or stigmasterol in CHCl_3_ (10 mL) was added dropwise phenyl isocyanate in the ratio 1:4 and some drops of HCl (37% *w/w* in water). The reaction mixture was refluxed for 24 h and monitored by TLC (n-hex/EtOAc 3:1). The obtained solution was concentrated to dryness and the crude product was purified by CC.

#### 3.2.2. Synthesis of (25R)-5-en-Spirost-3β-yl Phenylcarbamate (**7**)

Compound **7** was obtained (0.80 g, 62% yield) following the general procedure for synthesis of carbamates. Diosgenin (1.0 g, 2.4 mmol) and isocyanate (1.05 mL, 9.6 mmol). CC (n-hex/EtOAc 3:1)

Compound **7**: white solid, m.p. 219-220 °C, Rf = 0.76 (n-hex/EtOAc 3:1). ^1^H NMR (400 MHz, CDCl_3_) (Figure S7) δ = 7.37 (2H, d, *J* = 8.0 Hz, H-2a), 7.29 (2H, dd, *J* = 8.6, 7.2 Hz, H-3a), 7.05 (1H, t, *J* = 7.3 Hz, H-4a), 6.58 (1H, s, NH), 5.40 (1H, dt, *J* = 4.2, 1.9 Hz, H-6), 4.60 (1H, tt, *J* = 11.5, 4.8 Hz, H-3), 4.42 (1H, q, *J* = 7.5 Hz, H-16), 3.48 (1H, ddd, *J* = 11.0, 4.5, 2.0 Hz, H-26 eq), 3.38 (1H, t, *J* = 11.0 Hz, H-26ax), 2.44 (1H, ddd, *J* = 13.1, 5.1, 2.2 Hz, H-4 eq), 2.34 (1H, td, *J* = 12.3, 2.8 Hz, H-4 ax), 1.05 (3H, s, H-19), 0.98 (3H, d, *J* = 7.0 Hz, H-27), 0.79 (6H, d, *J* = 5.9 Hz, H-18 and H-21). ^13^C NMR (101 MHz, CDCl_3_) (Figure S1) δ = 153.16 (NHCO), 139.77 (C-5), 138.17 (C-1a), 129.16 (C-3a), 123.39 (C-4a), 122.61 (C-6), 118.69 (C-2a), 109.42 (C-22), 80.95 (C-16), 74.97 (C-3), 66.98 (C-26), 62.22 (C-17), 56.58 (C-14), 50.07 (C-9), 41.76 (C-20), 40.41 (C-13), 39.87 (C-12), 38.58 (C-4), 37.09 (C-1), 36.86 (C-10), 32.19 (C-15), 31.98 (C-8), 31.56 (C-7), 31.53 (C-23), 30.44 (C-25), 28.95 (C-24), 28.21 (C-2), 20.98 (C-11), 19.50 (C-19), 17.28 (C-27), 16.44 (C-18), 14.67 (C-21). ESI-HRMS, calculated for C_34_H_48_NO_4_: 534.3583 [M+H]^+^ found: *m*/*z* 534.3638.

#### 3.2.3. Synthesis of 5-en-Stigmast-3β-yl Phenylcarbamate (**9**)

Compound **8** was obtained (0.47 g, 73% yield) following the general procedure for synthesis of carbamates. β-sitosterol (0.5 g, 1.2 mmol) and isocyanate (0.52 mL, 4.8 mmol). CC (n-hex/EtOAc 12:1)

Compound **8** white solid, m.p. 160-161 °C, Rf = 0.77 (n-hex/EtOAc 5:1). ^1^H NMR (400 MHz, CDCl_3_) (Figure S2) δ = 7.37 (2H, d, *J* = 7.5 Hz, H-2a), 7.29 (2H, d, *J* = 6.3 Hz, H-3a), 7.04 (1H, t, *J* = 7.1 Hz, H-4a), 6.54 (1H, s, NH), 5.40 (1H, s, H-6), 4.68-4.53 (1H, m, H-3), 0.92 (3H, d, *J* = 6.2 Hz, H-19), 0.88-0.77 (12H, m, H-21, H-26, H-27 and H-29), 0.67 (3H, s, H-18). ^13^C NMR (101 MHz, CDCl_3_) (Figure S2) δ = 153.24 (NHCO), 139.92 (C-5), 138.35 (C-1a), 129.19 (C-3a), 123.48 (C-4a), 122.92 (C-6), 118.92 (C-2a), 75.23 (C-3), 57.01 (C-14), 56.43 (C-17), 50.39 (C-9), 46.24 (C-24), 42.62 (C-13), 40.05 (C-12), 38.72 (C-4), 37.27 (C-1), 36.86 (C-10), 36.38 (C-20), 34.30 (C-22), 32.18 (C-7), 32.16 (C-8), 29.60 (C-25), 28.42 (C-16), 28.36 (C-2), 26.63 (C-23), 24.51 (C-15), 23.42 (C-28), 21.32 (C-11), 19.96 (C-26), 19.51 (C-27), 19.31 (C-19), 19.01 (C-21), 12.18 (C-29), 12.07 (C-18). ESI-HRMS, calculated for C_36_H_56_NO_2_: 534.4311 [M+H]^+^ found: *m*/*z* 534.4382.

#### 3.2.4. Synthesis of 5,22-dien-Stigmast-3β-yl Phenylcarbamate (**11**)

Compound **11** was obtained (0.90 g, 71% yield) following the general procedure for synthesis of carbamates. Stigmasterol (1.0 g, 2.4 mmol) and isocyanate (1.05 mL, 9.6 mmol). CC (n-hex/EtOAc 12:).

Compound **11** white solid, m.p. 191-193 °C, Rf = 0.36 (n-hex/EtOAc 3:1). ^1^H NMR (400 MHz, CDCl_3_) (Figure S3) δ = 7.37 (2H, d, *J* = 7.9 Hz, H-2a), 7.34-7.27 (2H, m, H-3a), 7.05 (1H, t, *J* = 7.2 Hz, H-4a), 6.53 (1H, s, NH), 5.41 (1H, d, *J* = 4.5 Hz, H-16), 5.16 (1H, dd, *J* = 15.1, 8.5 Hz, H-23), 5.03 (1H, dd, *J* = 15.1, 8.5 Hz, H-22), 4.70-4.51 (1H, m, H-3), 0.80 (6H, d, *J* = 6.3 Hz, H-26 and H-27), 0.70 (3H, s, H-18). ^13^C NMR (101 MHz, CDCl_3_) (Figure S3) δ = 153.26 (NHCO), 139.94 (C-5), 138.41 (C-1a), 138.37 (C-22), 129.71 (C-23), 129.20 (C-3a), 123.49 (C-4a), 122.91 (C-6), 118.95 (C-2a), 75.25 (C-3), 57.10 (C-14), 56.36 (C-17), 51.48 (C-24), 50.45 (C-9), 42.54 (C-13), 40.52 (C-20), 39.95 (C-12), 38.73 (C-4), 37.29 (C-1), 36.89 (C-10), 32.22 (C-8), 32.16 (C-7), 32.08 (C-25), 29.00 (C-16), 28.37 (C-2), 25.54 (C-28), 24.58 (C-15), 21.40 (C-11), 21.32 (C-21), 21.15 (C-26), 19.51 (C-27), 19.21 (C-19), 12.32 (C-29), 12.28 (C-18). ESI-HRMS, calculated for C_36_H_54_NO_2_: 532.4154 [M+H]^+^ found: *m*/*z* 532.4196.

#### 3.2.5. Synthesis of Mixture (25R)- 5α,6α-Epoxy-Spirostan-3β-ol (**12a**) and (25R)-5β,6β-Epoxy-Espirostan-3β-ol (**12b**)

Following a procedure previously described [58], a solution of diosgenin (5.0 g, 12.1 mmol) in CHCl_3_ (30 mL) was cooled in an ice bath. After 5 min, *m*-chloroperoxybenzoic acid (*m*-CPBA) (3.34 g, 19.4 mmol) was added in small portions. The reaction mixture was stirred at room temperature for 3 h and consumption of starting material was monitored by TLC (n-hex/EtOAc 3:1). At the end of reaction, CHCl_3_ (80 mL) was added and the diluted mixture transferred to a separation funnel. This solution was washed (3x) with Na_2_SO_3_ (5% solution *w/w*), Na_2_CO_3_ (sat. solution) and water, respectively. The organic layer was dried over anhydrous Na_2_SO_4_ and then concentrated to dryness. The crude product was purified by recrystallization from acetone. Compounds **12a**/**12b** were obtained as an epoxide mixture of α/β epimers at C5-C6 positions (4.92 g, 95% yield, **12a**:**12b** = 2:1). ESI-HRMS, calculated for C_27_H_43_O_4_: 431.3161 [M+H]+ found: *m*/*z* 431.3171. The signals assignment was carried out from the ^1^H and ^13^C NMR spectra in the **12a**/**12b** mixture:

Compound **12a**: Rf = 0.19 (n-hex/EtOAc 3:1). ^1^H NMR (400 MHz, CDCl_3_) (Figure S4) δ = 4.38 (1H, q, *J* = 7.5 Hz, H-16), 4.17-3.61 (1H, m, H-3), 3.46 (1H, ddd, *J* = 10.9, 4.5, 2.0 Hz, H-26 eq), 3.36 (1H, td, *J* = 10.9, 2.1 Hz, H-26 ax), 2.90 (1H, d, *J* = 4.4 Hz, H-6), 1.09-0.94 (6H, m, H-19 and H-27), 0.79-0.65 (6H, m, H-18 and H-21). ^13^C NMR (101 MHz, CDCl_3_) (Figure S4) δ = 109.22 (C-22), 80.59 (C-16), 68.63 (C-3), 66.81 (C-26), 66.83 (C-5), 63.67 (C-17), 62.09 (C-6), 61.84 (C-14), 56.61 (C-9), 42.52 (C-20), 41.54 (C-13), 40.27 (C-12), 39.81 (C-4), 37.17 (C-10), 34.96 (C-1), 32.37 (C-15), 31.61 (C-2), 31.34 (C-8), 31.05 (C-25), 30.25 (C-7), 29.43 (C-23), 28.78 (C-24), 21.74 (C-11), 20.46 (C-27), 17.10 (C-19), 16.26 (C-18), 14.45 (C-21). ESI-HRMS, calculated for C_27_H_43_O_4_: 431.3161 [M+H]^+^ found: *m*/*z* 431.3171.

Compound **12b**: ^1^H NMR (400 MHz, CDCl_3_) (Figure S4) δ = 4.38 (1H, q, *J* = 7.5 Hz, H-16), 3.73-3.66 (1H, m, H-3), 3.46 (1H, ddd, *J* = 10.9, 4.5, 2.0 Hz, H-26 eq), 3.36 (1H, td, *J* = 10.9, 2.1 Hz, H-26 ax), 3.06 (1H, d, *J* = 2.3 Hz, H-6), 1.09-0.94 (6H, m, H-19 and H-27), 0.79-0.65 (6H, m, H-18 and H-21). ^13^C NMR (101 MHz, CDCl_3_) (Figure S4) δ = 109.26 (C-22), 80.64 (C-16), 69.39 (C-3), 66.81 (C-26), 65.61 (C-5), 63.67 (C-17), 62.86 (C-6), 61.84 (C-14), 55.95 (C-9), 42.18 (C-20), 41.58 (C-13), 40.22 (C-12), 39.84 (C-4), 37.17 (C-10), 34.99 (C-1), 32.72 (C-15), 31.71 (C-2), 31.32 (C-8), 31.01 (C-25), 30.27 (C-7), 29.29 (C-23), 28.75 (C-24), 21.74 (C-11), 20.46 (C-27), 17.07 (C-19), 16.15 (C-18), 15.96 (C-21).

#### 3.2.6. Synthesis of (25R)-Spirostan-3β,5α,6β-Triol (**13**)

Following a procedure previously described [59,60], the mixture **12a**/**12b** was cooled in an ice bath. After 5 min, HClO_4_ (3.9 mL, 65.1 mmol) was added dropwise. The reaction mixture was stirred at room temperature for 3 h and followed by TLC (EtOAc). Next, the solution was concentrated to one-third of the starting volume and the concentrated solution was poured over water. The resulting suspension was filtered and the obtained solid dried in a desiccator. Compound **13** was obtained (3.25 g, 78%) without further purification.

Compound **13**: white solid, m.p. 285-286 °C, Rf = 0.13 (EtOAc). ^1^H NMR (400 MHz, DMSO-d_6_) (Figure S5) δ = 4.43 (1H, s, C6-OH), 4.26 (1H, q, *J* = 7.8 Hz, H-16), 4.16 (1H, d, *J* = 5.6 Hz, C3-OH), 3.79 (1H, dt, *J* = 16.2, 5.5 Hz, H-3), 3.65 (1H, s, C5-OH), 3.40 (1H, dd, *J* = 9.9, 2.8 Hz, H-26 eq), 3.30 (1H, d, *J* = 3.8 Hz, H-6), 3.20 (1H, t, *J* = 11.0 Hz, H-26 ax), 1.03 (3H, s, H-19), 0.89 (3H, d, *J* = 6.9 Hz, H-27), 0.77-0.66 (6H, m, H-18 and H-21). ^13^C NMR (101 MHz, DMSO) (Figure S5) δ = 108.36 (C-22), 80.23 (C-16), 74.27 (C-5), 74.04 (C-6), 65.89 (C-3), 65.70 (C-26), 61.91 (C-17), 55.51 (C-14), 44.63 (C-9), 41.10 (C-20), 40.86 (C-4), 40.17 (C-13), 37.89 (C-10), 34.66 (C-7), 32.01 (C-15), 31.45 (C-1), 31.08 (C-8), 30.88 (C-2), 29.81 (C-23), 29.66 (C-25), 28.48 (C-24), 20.52 (C-11), 17.07 (C-27), 16.27 (C-19), 16.24 (C-18), 14.61 (C-21). ESI-HRMS, calculated for C_27_H_45_O_5_: 449.3267 [M+H]^+^ found: *m*/*z* 449.3325.

#### 3.2.7. Synthesis of (25R)-3β,5α-Dihydroxy-Espirostan-6-ona (**14**)

Compound **13** (2.0 g, 4.6 mmol) was dissolved in 160 mL of a 1,4-dioxane/water (7:1) mixture. Then, NBS (3.36 g, 18.9 mmol) was added at room temperature under no light conditions. The reaction mixture was stirred for 2 h and monitored by TLC (EtOAc). After this time, Na_2_SO_3_ 10% *w/w* was added and the mixture extracted with CHCl_3_. The organic layer was washed with water, dried over anhydrous Na_2_SO_4_ and then concentrated to dryness. Compound **14** was obtained (1.7 g, 83%) without further purification.

Compound **14**: white solid, m.p. 271-272 °C, Rf = 0.46 (EtOAc). ^1^H NMR (400 MHz, DMSO-d_6_) (Figure S6) δ = 5.32 (1H, s, C5-OH), 4.34 (1H, d, *J* = 5.6 Hz, C3-OH), 4.28 (1H, q, *J* = 7.4 Hz, H-16), 3.75-3.62 (1H, m, H-3), 3.45-3.36 (1H, m, H-26 eq), 3.20 (1H, t, *J* = 11.0 Hz, H-26 ax), 2.68 (1H, t, *J* = 12.3 Hz, H-7 ax), 0.90 (3H, d, *J* = 6.8 Hz, H-27), 0.73 (3H, d, *J* = 6.3 Hz, H-21), 0.68 (6H, d, *J* = 9.3 Hz, H-18 and H-19). ^13^C NMR (101 MHz, DMSO) (Figure S6) δ = 212.09 (C-6), 108.37 (C-22), 80.02 (C-5), 79.02 (C-16), 65.90 (C-3), 65.16 (C-26), 61.67 (C-17), 55.55 (C-14), 43.83 (C-9), 41.96 (C-10), 41.45 (C-20), 41.04 (C-7), 40.58 (C-13), 39.19 (C-4), 36.31 (C-12), 35.72 (C-8), 31.10 (C-15), 30.87 (C-2), 30.44 (C-1), 29.78 (C-23), 29.56 (C-25), 28.45 (C-24), 20.81 (C-11), 17.06 (C-27), 16.10 (C-18), 14.58 (C-19), 13.64(C-21). ESI-HRMS, calculated for C_27_H_43_O_5_: 447.3110 [M+H]^+^ found: *m*/*z* 447.3177.

#### 3.2.8. Synthesis of (25R)- 5α- Hydroxy-6-oxo-Spirostan-3β-yl Phenylcarbamate (**15**)

Compound **15** was obtained (0.86 g, 68% yield) following the general procedure for synthesis of carbamates. Compound **14** (1.0 g, 2.2 mmol) and isocyanate (1.12 mL, 8.8 mmol). CC (n-hex/EtOAc 3:1).

Compound **15** white solid, m.p. 264-266 ºC, Rf = 0.32 (n-hex/EtOAc 3:1). ^1^H NMR (400 MHz, CDCl_3_) (Figure S7) δ = 7.32 (2H, d, *J* = 7.7 Hz, H-2a), 7.29 (2H, d, *J* = 7.1 Hz, H-3a), 7.09-7.00 (1H, m, H-4a), 6.60 (1H, s, NH), 5.04 (1H, tt, *J* = 10.7, 5.0 Hz, H-3), 4.41 (1H, q, *J* = 7.8, 6.3 Hz, H-16), 3.47 (1H, ddd, *J* = 11.0, 4.6, 1.9 Hz, H-26 eq), 3.36 (1H, t, *J* = 10.9 Hz, H-26 ax), 2.90 (1H, s, C5-OH), 2.81-2.68 (1H, m, H-7 ax), 0.97 (3H, d, *J* = 6.8 Hz, H-27), 0.84 (3H, s, H-19), 0.79 (3H, d, *J* = 6.3 Hz, H-21), 0.75 (3H, s, H-18). ^13^C NMR (101 MHz, CDCl_3_) (Figure S7) δ = 211.98 (C-6), 153.39 (NHCO), 137.83 (C-1a), 129.19 (C-3a), 123.69 (C-4a), 119.00 (C-2a), 109.42 (C-22), 80.64 (C-5), 80.48 (C-16), 71.70 (C-3), 67.00 (C-26), 62.18 (C-17), 56.20 (C-14), 44.48 (C-9), 42.65 (C-10), 41.96 (C-20) 41.76 (C-7), 41.22 (C-13), 39.71 (C-12), 36.92 (C-4), 32.96 (C-8), 31.70 (C-15), 31.49 (C-23), 30.42 (C-1), 29.70 (C-25), 28.93 (C-24), 26.76 (C-2), 21.37 (C-11), 17.27 (C-19), 16.56 (C-27), 14.59 (C-18), 14.18 (C-21). ESI-HRMS, calculated for C_34_H_48_NO_6_: 566.3481 [M+H]^+^ found: *m*/*z* 566.3527.

#### 3.2.9. Synthesis of Mixture (25R)-5α,6α-epoxy-Spirostan-3β-yl Phenylcarbamate (**16a**) and (25R)- 5β,6β-Epoxy-Spirostan-3β-yl Phenylcarbamate (**16b**)

A solution of compound **7** (0.1 g, 0.19 mmol) in CHCl_3_ (15 mL) was placed in an ice bath. After 5 min, *m*-CPBA (53 mg, 0.30 mmol) was added. The reaction mixture was stirred at room temperature for 3 h based on TLC (n-hex/EtOAc 3:1) consumption of starting material. Next, CHCl_3_ (55 mL) was added and the diluted mixture transferred to a separation funnel. This solution was washed (3x) with Na_2_SO_3_ (5% solution *w/w*), Na_2_CO_3_ (sat. solution) and water, respectively. The organic layer was dried over anhydrous Na_2_SO_4_ and then concentrated to dryness. The crude product was purified by CC (n-hex/EtOAc 6:1). Compounds **16a** and **16b** were obtained as a mixture of α/β epimers at C5-C6 positions (0.1 g, 94% yield, **16a**:**16b** = 3:1). ESI-HRMS, calculated for C_34_H_48_NO_5_: 550.3532 [M+H]^+^ found: *m*/*z* 550.3567. The signals assignment was carried out from the ^1^H and ^13^C NMR spectra in the **16a**/**16b** mixture (Figure S8).

Compound **16a**: Rf = 0.59 (n-hex/EtOAc 3:1). ^1^H NMR (400 MHz, CDCl_3_) (Figure S8) δ = 7.33 (4H, dd, *J* = 16.6, 9.1 Hz, H-2a and H-3a), 7.11-6.98 (1H, m, H-4a), 6.55 (1H, s, NH), 5.04-4.68 (1H, m, H-3), 4.38 (1H, q, *J* = 7.3 Hz, H-16), 3.53-3.41 (1H, m, H-26 eq), 3.36 (1H, td, *J* = 10.8, 2.2 Hz, H-26 ax), 2.91 (1H, d, *J* = 5.7 Hz, H-6), 1.07 (3H, s, H-19), 0.95 (3H, d, *J* = 6.8 Hz, H-27), 0.78 (3H, d, *J* = 6.2 Hz, H-21), 0.74 (3H, s, H-18). ^13^C NMR (101 MHz, CDCl_3_) (Figure S8) δ = 152.95 (NHCO), 138.29 (C-1a), 129.19 (C-3a), 123.37 (C-4a), 118.94 (C-2a), 109.38 (C-22), 80.83 (C-16), 72.62 (C-3), 67.08 (C-26), 65.26 (C-5), 62.36 (C-17), 59.12 (C-6), 56.88 (C-14), 42.82 (C-9), 41.91 (C-20), 40.59 (C-13), 39.67 (C-12), 36.68 (C-4), 35.41 (C-10), 32.45 (C-1), 31.91 (C-15), 31.71 (C-8), 30.54 (C-23), 29.80 (C-7), 29.25 (C-25), 29.12 (C-24), 27.84 (C-2), 20.73 (C-11), 17.24 (C-27), 16.42 (C-19), 16.16 (C-18), 14.59 (C-21).

Compound **16b**: ^1^H NMR (400 MHz, CDCl_3_) (Figure S8) δ = 7.33 (4H, dd, *J* = 16.6, 9.1 Hz, H-2a and H-3a), 7.11-6.98 (1H, m, H-4a), 6.58 (1H, s, NH), 4.82-4.74 (1H, m, H-3), 4.38 (1H, q, *J* = 7.3 Hz, H-16), 3.53-3.41 (1H, m, H-26 eq), 3.36 (1H, td, *J* = 10.8, 2.2 Hz, H-26 ax), 3.11 (1H, d, *J* = 1.7 Hz, H-6), 1.07 (3H, s, H-19), 0.95 (3H, d, *J* = 6.8 Hz, H-27), 0.78 (3H, d, *J* = 6.2 Hz, H-21), 0.74 (3H, s, H-18). ^13^C NMR (101 MHz, CDCl_3_) (Figure S8) δ = 152.98 (NHCO), 137.98 (C-1a), 129.03 (C-3a), 123.52 (C-4a), 118.88 (C-2a), 109.22 (C-22), 80.73 (C-16), 72.37 (C-3), 66.92 (C-26), 63.49 (C-5), 62.49 (C-17), 58.97 (C-6), 56.09 (C-14), 42.65 (C-9), 41.75 (C-20), 40.37 (C-13), 39.94 (C-12), 36.67 (C-4), 35.35 (C-10), 32.79 (C-1), 31.85 (C-15), 31.56 (C-8), 30.73 (C-23), 29.42 (C-7), 29.09 (C-25), 28.96 (C-24), 27.61 (C-2), 21.79 (C-11), 17.08 (C-27), 16.26 (C-19), 15.99 (C-18), 14.42 (C-21).

#### 3.2.10. Synthesis of ((25R)-5α-Hydroxy-6-Hydroxymino-3β-yl Phenylcarbamate (**17**)

Compound **15** (0.2 g, 0.4 mmol) was dissolved in ethanol (20 mL). Then, NH_2_OH·HCl (0.11 g, 1.6 mmol) and NaOAc·3H_2_O (0.22 g, 1.6 mmol) were added. The resulting mixture was refluxed for 3 h while been monitored by TLC (n-hex/EtOAc 3:1). The solvent volume was reduced to one-third of the initial volume and the concentrated solution was poured over water. The formed solid was filtered and purified by CC (n-hex/EtOAc 3:1). Compound **17** was obtained (0.18 g, 77%).

Compound **17** white solid, m.p. 257-259 °C, Rf = 0.36 (n-hex/EtOAc 3:1). ^1^H NMR (400 MHz, DMSO-d_6_) (Figure S9) δ = 10.51 (1H, s, N-OH), 9.50 (1H, s, NH), 7.45 (2H, d, *J* = 7.9 Hz, H-2a), 7.25 (2H, t, *J* = 7.8 Hz, H-3a), 6.96 (1H, t, *J* = 7.3 Hz, H-4a), 5.09-4.97 (2H, m, H-3 and C5-OH), 4.30 (1H, q, *J* = 7.1 Hz, H-16), 3.46-3.37 (1H, m, H-26 eq), 3.22 (1H, t, *J* = 11.0 Hz, H-26 ax), 2.98 (1H, dd, *J* = 13.3, 4.6 Hz, H-7 ax), 0.91 (3H, d, *J* = 6.8 Hz, H-27), 0.81-0.67 (9H, m, H-18, H-19 and H-21). ^13^C NMR (101 MHz, DMSO) (Figure S9) δ = 159.16 (C-6), 153.10 (NHCO), 139.30 (C-3a), 128.65 (C-4a), 122.13 (C-2a), 118.06 (C-22), 108.41 (C-16), 80.13 (C-5), 75.42 (C-3), 70.92 (C-26), 65.91 (C-17), 61.65 (C-14), 55.47 (C-9), 44.22 (C-10), 41.07 (C-20), 40.56 (C-4), 40.40 (C-13), 34.61 (C-12), 33.98 (C-8), 31.22 (C-15), 30.84 (C-1), 29.81 (C-23), 29.35 (C-25), 28.45 (C-7), 26.83 (C-24), 24.66 (C-2), 20.76 (C-11), 17.05 (C-19), 16.19 (C-27), 14.57 (C-18), 14.02 (C-21). ESI-HRMS, calculated for C_34_H_49_N_2_O_6_: 581.3590 [M+H]^+^ found: *m*/*z* 581.3623.

#### 3.2.11. Synthesis of (25R)-5α,6β-Dihydroxy-Spirostan-3β-yl Phenylcarbamate (**18**)

A solution of compound **15** (1.0 g, 1.8 mmol) in ethanol (85 mL) was placed in an ice bath and NaBH_4_ (0.1 g, 2.7 mmol) dissolved in EtOH (5 mL) was added under stirring. Reaction was stopped after 30 min when TLC (n-hex/EtOAc 3:1) showed no more starting material. Next, reaction crude was poured over water and the resulting suspension filtered. The solid was purified by CC (n-hex/EtOAc 4:1). Compound **18** was obtained (0.85 g, 83% yield).

Compound **18** white solid, m.p. 258-260 °C, Rf = 0.21 (n-hex/EtOAc 3:1). ^1^H NMR (400 MHz, DMSO-d_6_) (Figure S10) δ = 9.49 (1H, s, NH), 7.45 (2H, d, *J* = 8.0 Hz, H-2a), 7.25 (2H, dd, *J* = 8.5, 7.3 Hz, H-3a), 6.96 (1H, t, *J* = 7.3 Hz, H-4a), 5.08 (1H, tt, *J* = 11.0, 5.4 Hz, H-3), 4.58 (1H, d, *J* = 4.2 Hz, C6-OH), 4.28 (1H, q, *J* = 13.4, 6.3 Hz, H-16), 4.03 (1H, s, C5-OH), 3.45-3.36 (2H, m, H-6 and H-26 eq), 3.21 (1H, t, *J* = 11.0 Hz, H-26 ax), 1.09 (3H, s, H-19), 0.90 (3H, d, *J* = 6.9 Hz, H-27), 0.77-0.70 (6H, m, H-18 and H-21). ^13^C NMR (101 MHz, DMSO) (Figure S10) δ = 153.19 (NHCO), 139.36 (C-1a), 128.63 (C-3a), 122.08 (C-4a), 118.02 (C-2a), 108.37 (C-22), 80.20 (C-16), 74.24 (C-5), 73.63 (C-6), 71.27 (C-3), 66.92 (C-26), 65.88 (C-17), 61.84 (C-14), 55.41 (C-9), 54.88 (C-20), 44.55 (C-13), 41.10 (C-12), 37.87 (C-10), 37.14 (C-4), 34.51 (C-7), 31.65 (C-8), 31.43 (C-15), 30.85 (C-1), 29.80 (C-23), 29.59 (C-25), 28.46 (C-24), 27.07 (C-2), 20.47 (C-11), 17.07 (C-27), 16.25 (C-19), 16.19 (C-18), 14.60 (C-21). ESI-HRMS, calculated for C_34_H_50_NO_6_: 568.3638 [M+H]^+^ found: *m*/*z* 568.3681.

### 3.3. Rice-Lamina Inclination Assay

The biological activity of the compounds was evaluated by the tilt test of the sheet according to the procedure described [30]. After soaking the rice seeds (Oryza sativa) Zafiro variety provided by the Institute of Agricultural Research (INIA-Quilamapu) in sterile distilled water for 24 h, the seeds were selected and cultivated at 22 °C in a plant growing chamber growing under a photoperiod of 16 h of light/8 h of darkness in pots with soil and plenty of water. Etiolated rice seedlings were grown so far, which were homogeneous and presented the second internode of the rice blade for cutting, this segment was deposited in sterile distilled water in Petri dishes for 24 h. Subsequently, six segments per treatment were incubated in a Petri dish containing 60 mL of distilled water and compounds **7**, **9**, **11**, **15**, **17**, **18** and mixture **16a**/**16b** at concentrations 0.01, 0.1 and 1 µM, for 48 h in the dark. Segments incubated with **1** under the same conditions were used as positive control, whereas sterile distilled water was added to segments used as negative control. Finally, the magnitude of the angle induced between the blade and the sheath was measured. Two bioassays were evaluated.

### 3.4. Molecular Docking

#### 3.4.1. Ligand/Molecular Target Selection and Preparation

2D structures of the steroids for the study were obtained using ChemDraw Professional 15.0 (Perkin Elmer, Waltham, MA, USA). Three-dimensional structure coordinates were generated and preliminarily optimized with the MM2 force-field—Steepest Descent Algorithm [61] implemented in Chem3D 15.0 (Perkin Elmer). All structures were further optimized through PM7 semi-empirical method implemented in MOPAC 2016 code (http://OpenMOPAC.net; accessed on 1–10 July 2020). All ligand PDB files were converted into PDBQT format (input for AutoDock Vina) [52]. The charges on the ligand atoms were generated using the Gasteiger model, nonpolar hydrogens were merged, and default rotatable bonds were retained using TORSDOF utility in AutoDockTools (http://mgltools.scripps.edu; accessed on 2 February 2020) [62].

The crystal structure of the protein Brassinosteroid Insensitive 1 (BRI1) in complex with BRI1-Associated Receptor Kinase 1 (BAK1) and the natural ligand **1**, PDB ID: 4m7e, resolved at 3.60 Å was retrieved from Protein Data Bank (http://www.rcsb.org; accessed on 2–15 January 2019). The structure was optimized using pdb2pqr.py (Version 2.0.0) implemented in the web server PDB2PQR (http://nbcr-222.ucsd.edu/pdb2pqr_2.0.0/; accessed on 3–22 February 2019), using the AMBER force-field, and the protonation state of ionizable groups at pH 8 was assigned using PROPKA [58,59]. The grid search was selected of size 22 × 22 × 22 Å [62] with the center of simulation box matching the center of 1.

#### 3.4.2. Docking Procedure and Analysis of Protein-Ligand Complexes.

Docking simulations were performed using AutoDock Vina 1.1.2. The active site of the proteins heterodimer was kept rigid, and non-flexible docking was carried out. The docking parameters were set to default except the following: exhaustiveness = 32 and num_modes = 2. The Vina code predicts the adopted conformations with the binding affinity (kcal/mol). The best two docked conformations, according to the binding affinity from 15 independent runs, were analyzed to produce 30 final docked poses. These poses were clustered according to the Root-Mean-Square-Deviation (RMSD), with a cut-off of 1.5 Å among poses. The binding energy of the cluster is the average binding energy of all the conformations present in it. The cluster with the lowest binding energy and higher number of conformations was selected as the representative binding mode of that particular ligand. Graphical representations of the protein-ligand complexes (Figure S11) were prepared using PyMol^TM^ version 2.1.0 (Schrödinger, New York, NY, USA, https://pymol.org/; accessed on 10 February 2020).

## 4. Conclusions

In this study we reported the synthesis of seven new *N*-phenyl carbamates BRs analogs, starting from natural diosgenin, stigmasterol, β-sitosterol and synthetic hydroxysteroids. Several functional groups such as double bonds, epoxy, hydroxy, keto, and oxime were combined with the carbamate moiety in the same chemical structure. A rice-lamina inclination assay was conducted for all compounds demonstrating their effect as plant-growth enhancers. Among the five diosgenin-derived carbamates, compounds **15** with a keto group in C6, and mixture of epoxides **16a**/**16b** (α and β isomers) were more active in vitro than compounds **7**, **17** and **18**, which carry double bond, hydroxyl or hydroxymino group in positions C5 and/or C6, respectively. The mixture of epoxides, **16a**/**16b**, showed activity similar to that of natural brassinolide. Finally, a docking study showed essential aspects related to the interaction of carbamates and BRI1-BAK1, BRs receptor proteins in *Arabidopsis thaliana*.

## Figures and Tables

**Table 1:** Effect of brassinolide (**1**) and BRs analogs **7**, **9**, **11**, **15**, **16a**/**16b**, **17** and **18**, on the lamina inclination test of rice seedlings.

BRs Analogs	Inclination between Laminae and Sheaths (Degrees ± Standard Error)		
Number	Concentration (μM)		
	0.01	0.10	1.0
**7**	21 ± 3 ^a^	16 ± 5 ^c^	21 ± 3 ^a^
**9**	35 ± 3 ^b^	30 ± 3 ^b^	11 ± 5 ^c^
**11**	31 ± 3 ^b^	31 ± 3 ^b^	14 ± 1 ^c^
**15**	29 ± 1 ^b^	33 ± 3 ^b^	40 ± 3 ^b^
**16a/16b**	25 ± 3 ^a^	51 ± 1 ^d^	41 ± 1 ^b^
**17**	13 ± 5 ^c^	36 ± 5 ^b^	38 ± 2 ^b^
**18**	13 ± 5 ^c^	29 ± 1 ^b^	29 ± 1 ^b^
**1** (C+)	34 ± 6 ^b^	55 ± 8 ^d^	78 ± 9 ^e^
C- (water)	11 ± 6 ^c^		

These values represent the mean ± standard deviation of two independent experiments with at least six replicates each (n = 12). Values with different superscript letters are statistically different (*p* < 0.05). Post-Hoc Tukey HSD test was applied to show statistically significant differences among the means.

## Data Availability

The data presented in this study are available in Supplementary Material.

## Supplementary Materials

The following are available online at https://www.mdpi.com/1422-0067/22/5/2330/s1, Figure S1: NMR spectra of (25*R*)-spirost-3-*N*-phenyl-carbamoyl-5-en-3β-ol (**7**); Figure S2: NMR spectra of 3-*N*-phenyl-carbamoyl-stigmast-5-en-3β-ol (**9**); Figure S3: NMR spectra of 3-*N*-phenyl-carbamoyl-stigmast-5,22-dien-3β-ol (**11**); Figure S4: NMR spectra of mixture (25*R*)-spirostan-5α,6α-epoxi-3β-ol (**12a**) and (25*R*)-spirostan-5β,6β-epoxi-3β-ol (**12b**); Figure S5: NMR spectra of (25*R*)-spirostan-3β,5α,6β-triol (**13**); **Figure S6**: NMR spectra of (25*R*)-espirostan-3β,5α-dihidroxi-6-ona (**14**); Figure S7: NMR spectra of (25*R*)-spirostan-3-N-phenyl-carbamoyl-3β,5α-dihydroxy-6-one (**15**); Figure S8: NMR spectra of mixture (25*R*)-spirostan-5α,6α-epoxi-3-*N*-phenyl-carbamoyl-3β-ol (**16a**) and (25*R*)-spirostan-5β,6β-epoxi-3-*N*-phenyl-carbamoyl-3β-ol (**16b**); Figure S9: NMR spectra of (25*R*)-espirostan-3-*N*-fenil-carbamoil-3β,5α-dihydroxy-6-oxime (**17**); Figure S10: NMR spectra of (25*R*)-spirostan-3-*N*-phenyl-carbamoyl-3β,5α,6β-triol (**18**); Figure S11: Representations of ligand-BRI1/BAK1 complexes. Crystallographic poses of carbamates steroids **7**, **9**, **11**, **12a**, **12b**, **13**, **14**, **15**, **16a**, **16b**, **17** and **18**. Crystallographic pose of **1** is in black sticks; Figure S12: Comparison between the interactions of heterodimer BRI1/BAK1 with **1** and steroidal carbamates ligands; Table S1: Energy (kcal/mol) and conformation clusters of the docked ligands.

## Abbreviations

BRI1	Brassinosteroid Insensitive 1
BAK1	BRI-Associated Kinase 1
BRs	Brassinosteroids
CC	Column Chromatography
TLC	Thin-Layer Chromatography
*m*-CPBA	*meta*-Chloroperoxybenzoic Acid
NBS	N-Bromosuccinimide
LRR-RLKs	Leucine-Rich Repeat Receptor-Like Kinases
SERK	Somatic Embryogenesis Receptor Kinase
NMR	Nuclear Magnetic Resonance
ppm	parts per millions
s	singlet
d	doublet
t	triplet
q	quartet
dd	double doublets
dt	double triplets
td	tripe doublets
ddd	doublet of double doublets
tt	triplet of triplets
m	multiplet
eq	equatorial H disposition
ax	axial H disposition
ESI-HRMS	High Resolution Electrospray Ionization Mass Spectrometry
*n*-hex	*n*-hexane
EtOAc	Ethyl acetate
THF	Tetrahydrofuran
DMSO	Dimethyl sulfoxide
PhNCO	phenyl isocyante
rt.	room temperature
