# Supplementary material for: Synthesis of New Steroidal Carbamates with Plant-Growth-Promoting Activity: Theoretical and Experimental Evidence
Source: Int J Mol Sci. 2021 Feb 26;22(5):2330. doi: 10.3390/ijms22052330 (PMC7956523; doi:10.3390/ijms22052330)
Supplement: Supplementary file 1 [file ijms-22-02330-s001.pdf]

Article

Synthesis of new steroidal carbamates with plant-growth-promoting activity: theoretical and experimental evidence

Daylin Fernández Pacheco[1], Leonardo González Ceballos[1], Armando Zaldo Castro[1], Marcos R. Conde González[1], Laura González de la Torre[2], Lia Pérez Rostgaard[1], Luis Espinoza[3*], Katy Díaz[3], Andrés F. Olea[4], and Yamilet Coll García[1*]

[1] Center for Natural Product Research, Faculty of Chemistry, University of Havana, Zapata and G, Havana 10400, Cuba; daylinfp@fq.uh.cu (D.F.P.); leo@fq.uh.cu (L.G.C.); zaldo@fq.uh.cu (A.Z.C.); mrconde@fq.uh.cu (M.R.C.G.); lrostgaard@estudiantes.fq.uh.cu (L.P.R.), yamcoll@fq.uh.cu (Y.C.G.)

[2] Sierra Maestra Science, Technology and Innovation Entity, Barlovento Complex, 5ta Ave. and 246, Havana, 11300, Cuba. ltorre@bionaturasm.cu (L.G.T.)

[3] Departamento de Química, Universidad Técnica Federico Santa María, Avenida España 1680, Valparaíso 2340000, Chile; katy.diaz@usm.cl (K.D.); luis.espinozac@usm.cl (L.E.)

[4] Instituto de Ciencias Químicas Aplicadas, Facultad de Ingeniería, Universidad Autónoma de Chile, El Llano Subercaseaux 2801, Santiago 8900000, Chile; andres.olea@uautonoma.cl (A.F.O.)

* Correspondence: yamcoll@fq.uh.cu (Y.C. G); luis.espinozac@usm.cl (L.E.); +56 32 2654225 (L.E.)

Received: date; Accepted: date; Published: date

Supplementary Materials: **The following are available online at www.mdpi.com/xxx/s1, Figure S1**: NMR spectra of (25*R*)-spirost-3-*N*-phenyl-carbamoyl-5-en-3β-ol (**7**); **Figure S2**: NMR spectra of 3-*N*-phenyl-carbamoyl-stigmast-5-en-3β-ol (**9**); **Figure S3**: NMR spectra of 3-*N*-phenyl-carbamoyl-stigmast-5,22-dien-3β-ol (**11**); **Figure S4**: NMR spectra of mixture (25*R*)-spirostan-5α,6α-epoxi-3β-ol (**12a**) and (25*R*)-spirostan-5β,6β-epoxi-3β-ol (**12b**); **Figure S5**: NMR spectra of (25*R*)-spirostan-3β,5α,6β-triol (**13**); **Figure S6**: NMR spectra of (25*R*)-espirostan-3β,5α-dihidroxi-6-ona (**14**); **Figure S7**: NMR spectra of (25*R*)-spirostan-3-N-phenyl-carbamoyl-3β,5α-dihydroxy-6-one (**15**); **Figure S8**: NMR spectra of mixture (25*R*)-spirostan-5α,6α-epoxi-3-*N*-phenyl-carbamoyl-3β-ol (**16a**) and (25*R*)-spirostan-5β,6β-epoxi-3-*N*-phenyl-carbamoyl-3β-ol (**16b**); **Figure S9**: NMR spectra of (25*R*)-espirostan-3-*N*-fenil-carbamoil-3β,5α-dihydroxy-6-oxime (**17**); **Figure S10**: NMR spectra of (25*R*)-spirostan-3-*N*-phenyl-carbamoyl-3β,5α,6β-triol (**18**); **Figure S11**: Representations of ligand-BRI1/BAK1 complexes. Crystallographic poses of carbamates steroids **7**, **9**, **11**, **12a**, **12b**, **13**, **14**, **15**, **16a**, **16b**, **17** and **18**. Crystallographic pose of **1** is in black sticks; **Figure S12**: Comparison between the interactions of heterodimer BRI1/BAK1 with **1** and steroidal carbamates ligands; **Table S1**: Energy (kcal/mol) and conformation clusters of the docked ligands.

Figure S1: NMR spectra of (25R)-5-en-spirost-3β-yl phenylcarbamate (**7**)

^{1}H-NMR

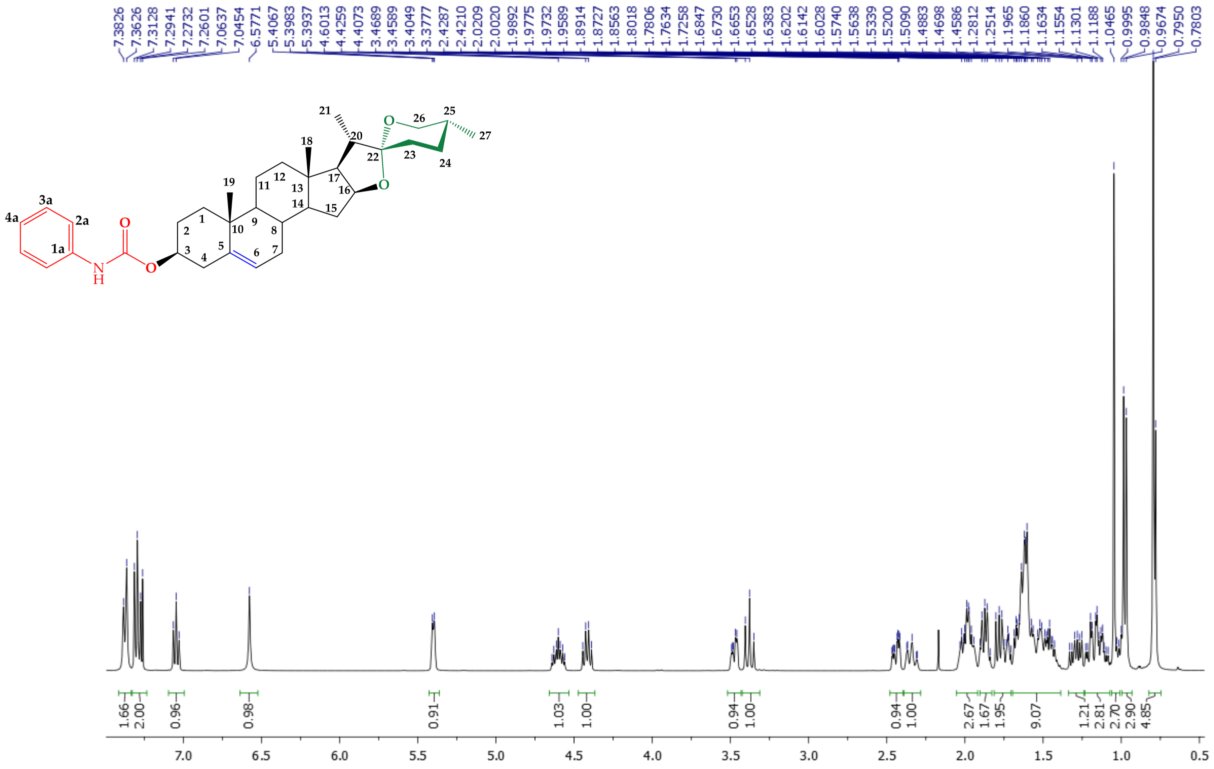

^{13}C-NMR

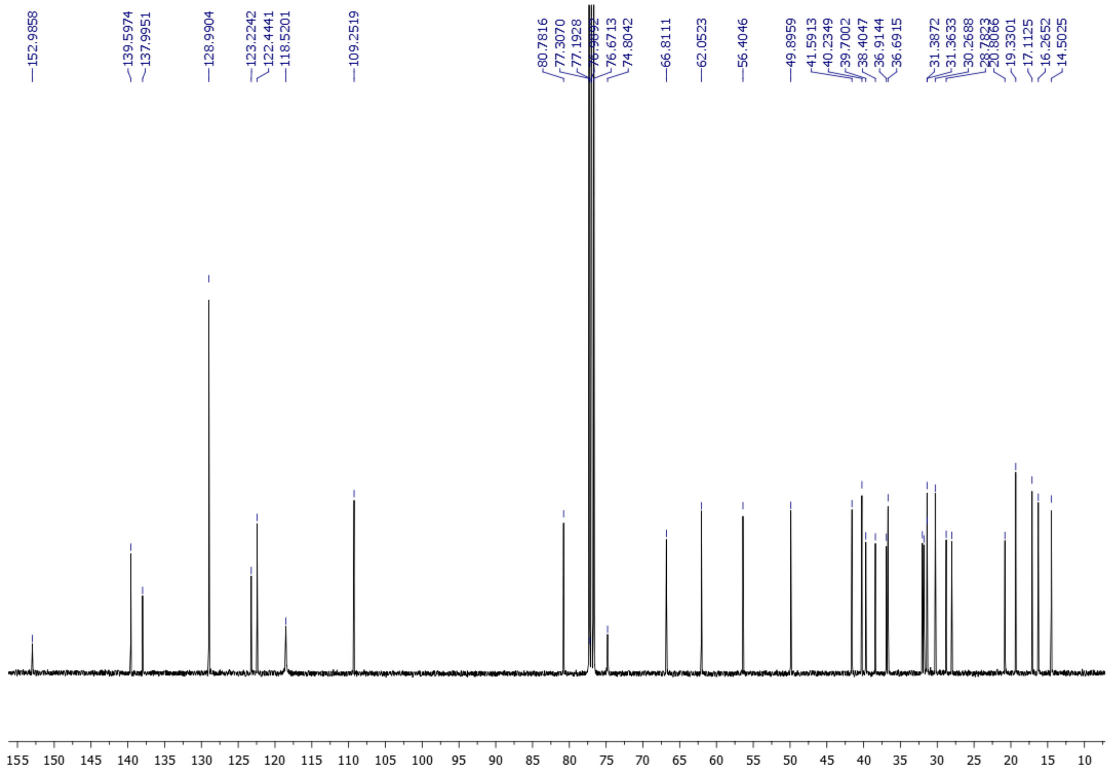

Figure S2: NMR spectra of Synthesis of 5-en-stigmast-3β-yl phenylcarbamate (**9**)

^{1}H-NMR

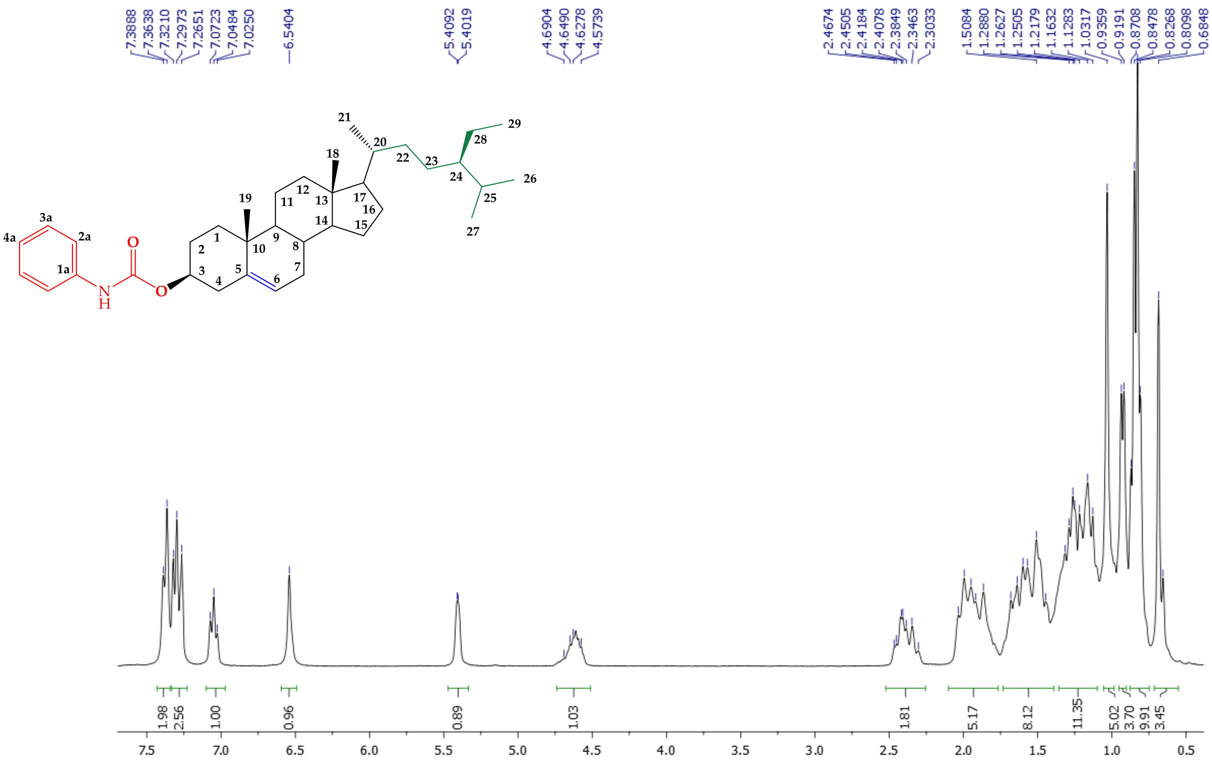

^{13}C-NMR

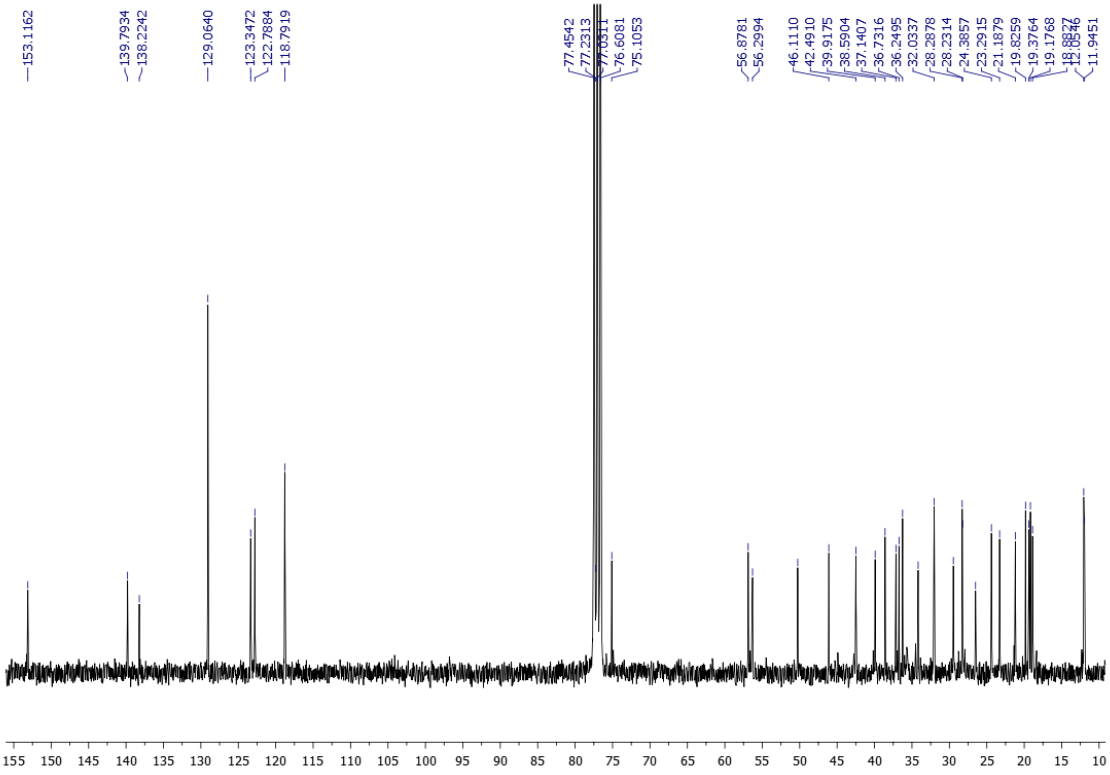

Figure S3: NMR spectra of 5,22-dien-stigmast-3β-yl phenylcarbamate (**11**)

^{1}H-NMR

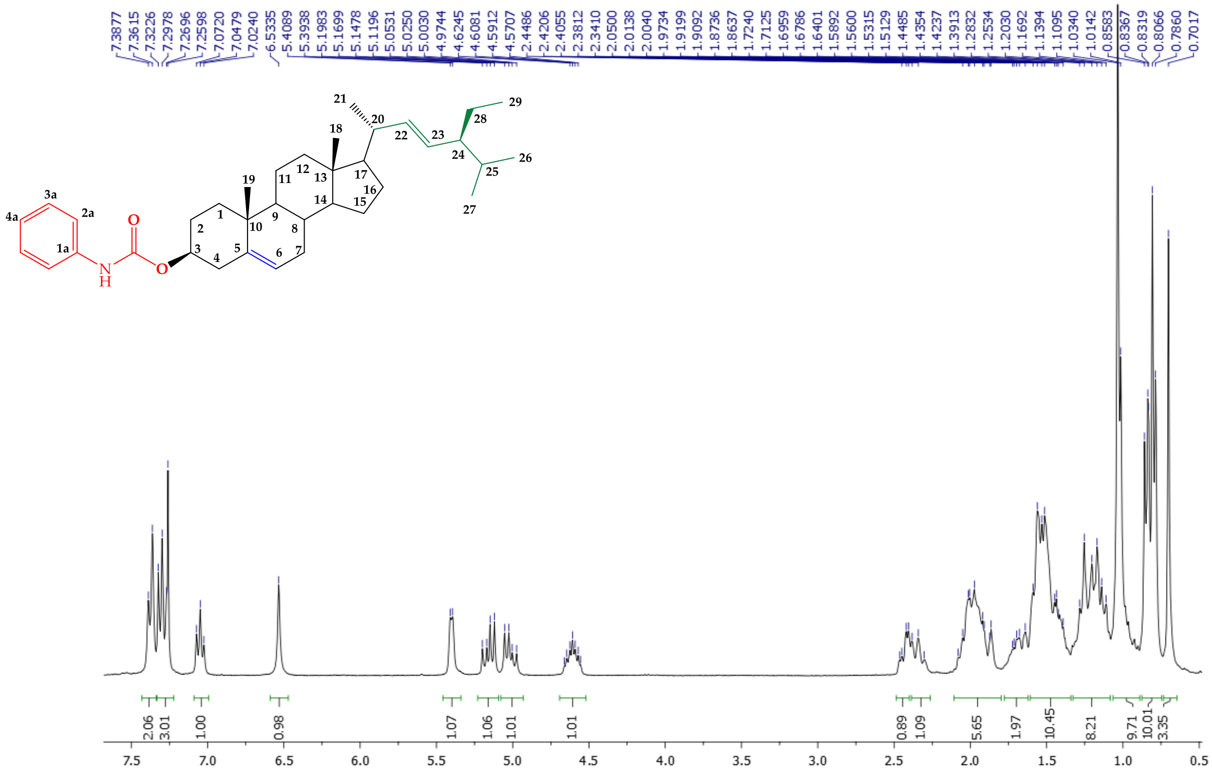

^{13}C-NMR

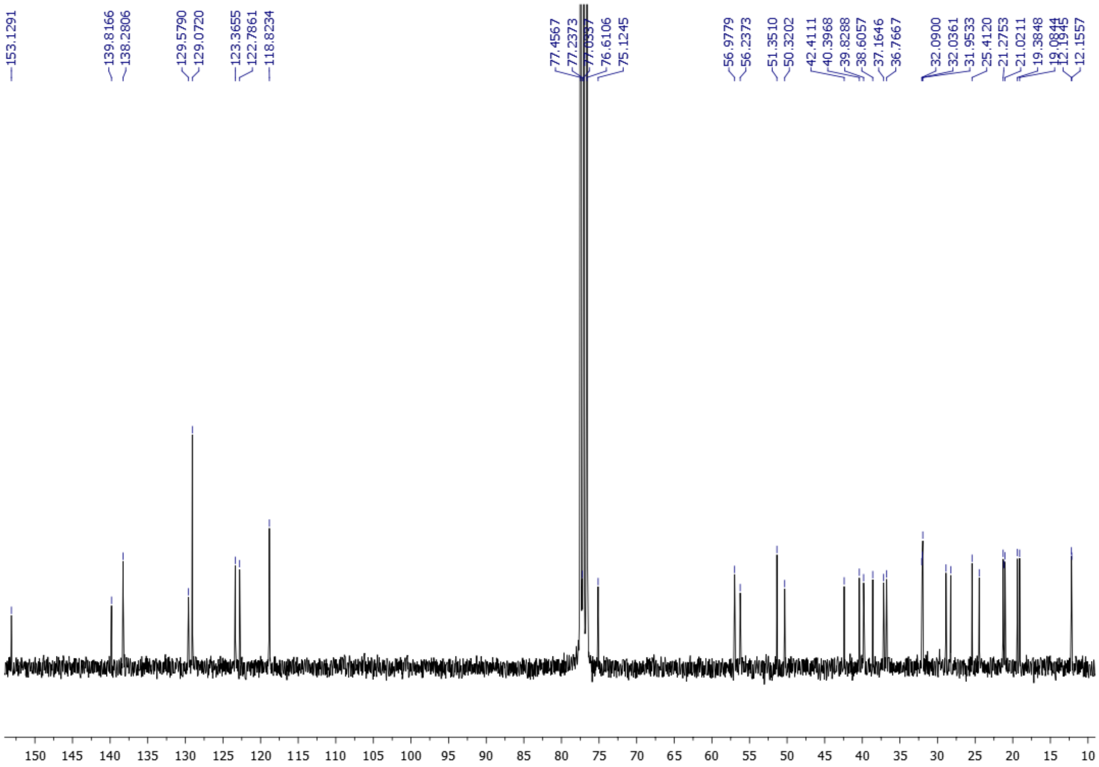

Figure S4: NMR spectra of mixture (25R)- 5α,6α-epoxy-spirostan-3β-ol (**12a**) and (25R)-5β,6β-epoxy-espirostan-3β-ol (**12b**)

^{1}H-NMR

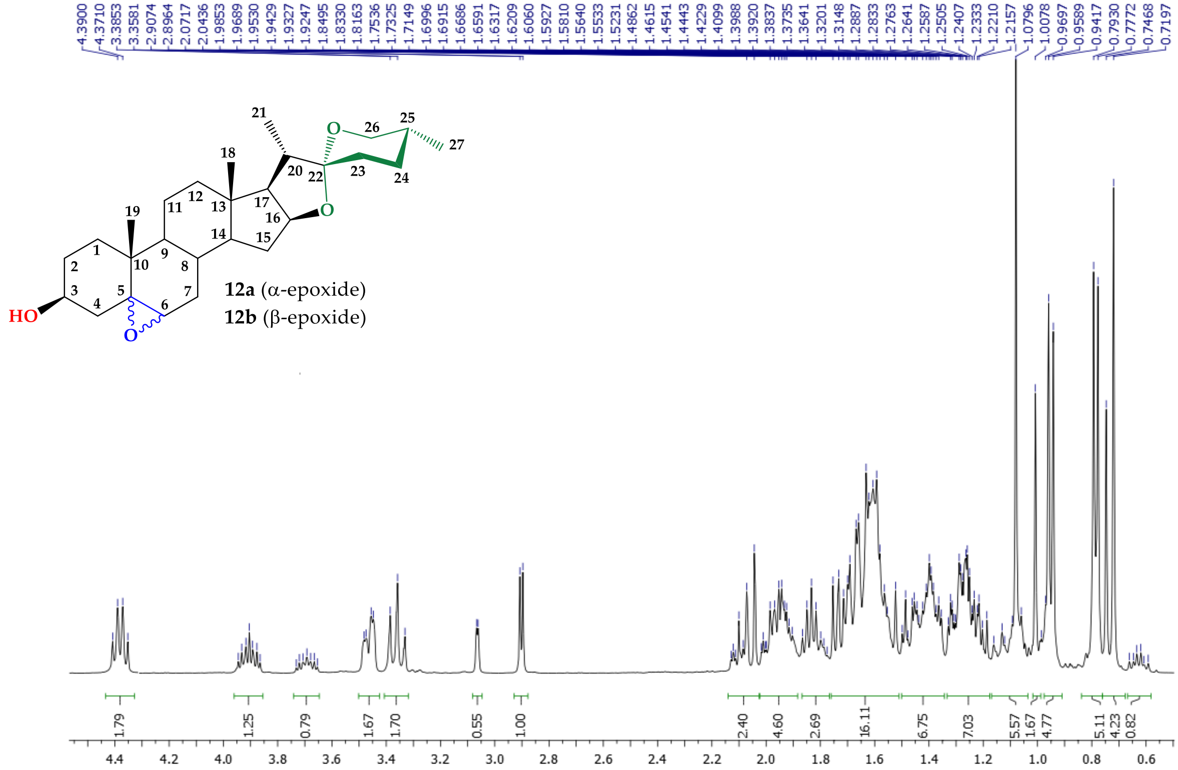

^{13}C-NMR

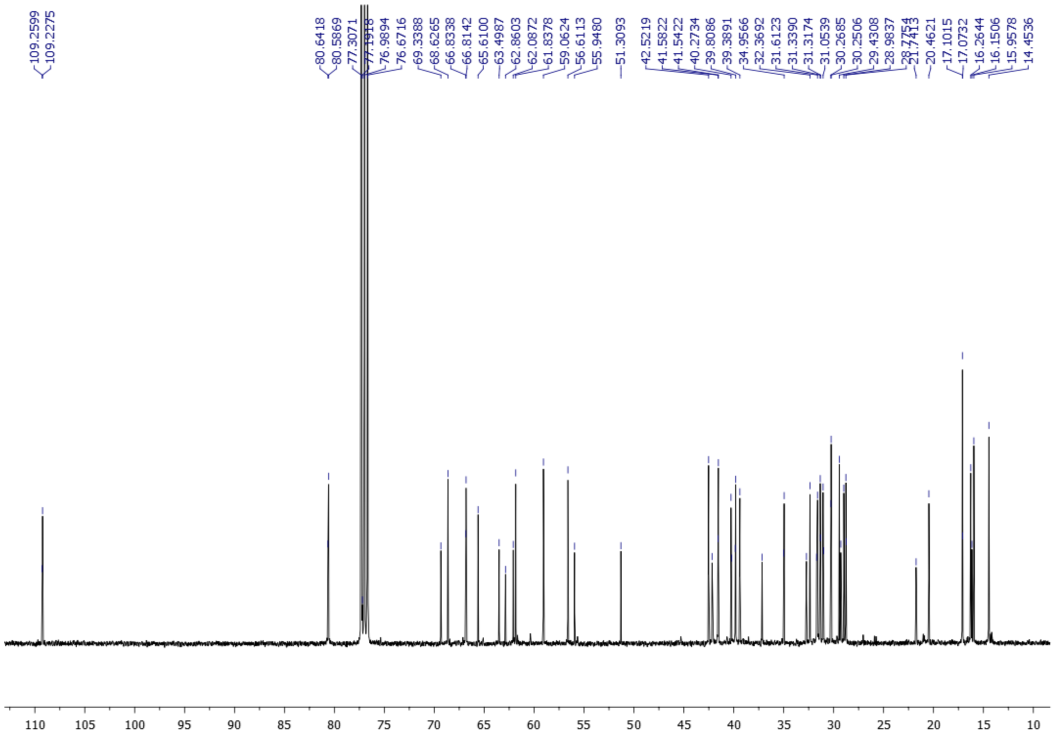

Figure S5: NMR spectra of (25R)-spirostan-3β,5α,6β-triol (**13**)

^{1}H NMR

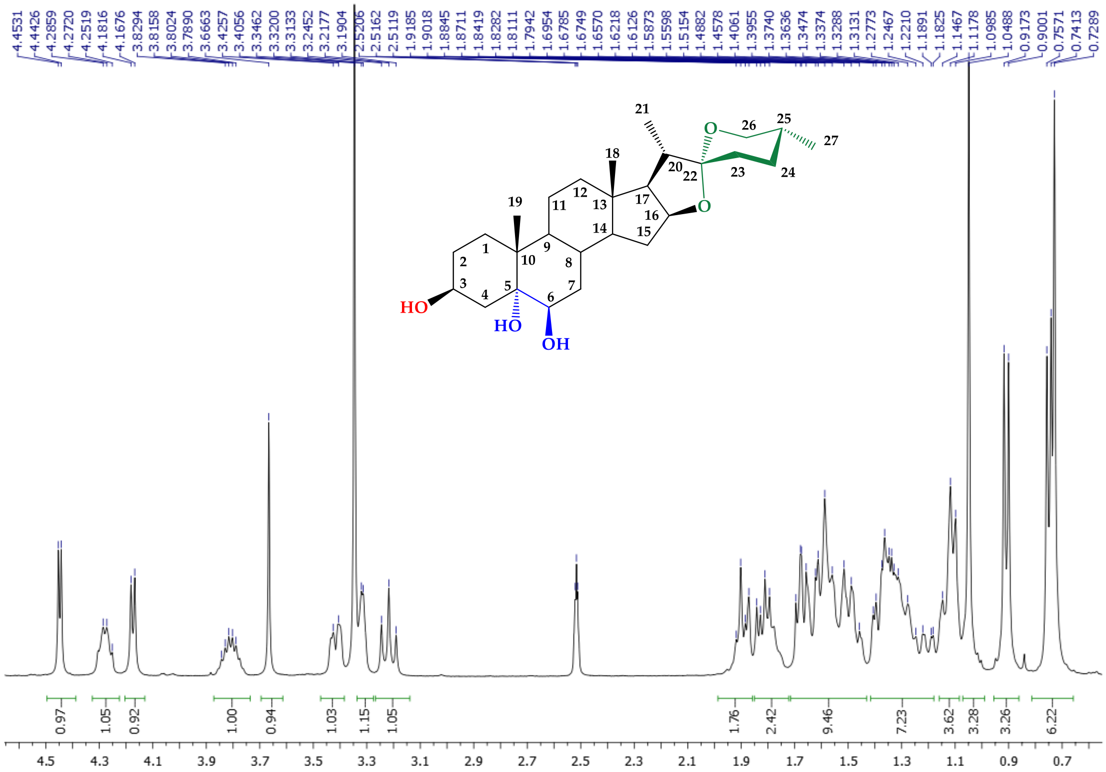

^{13}C-NMR

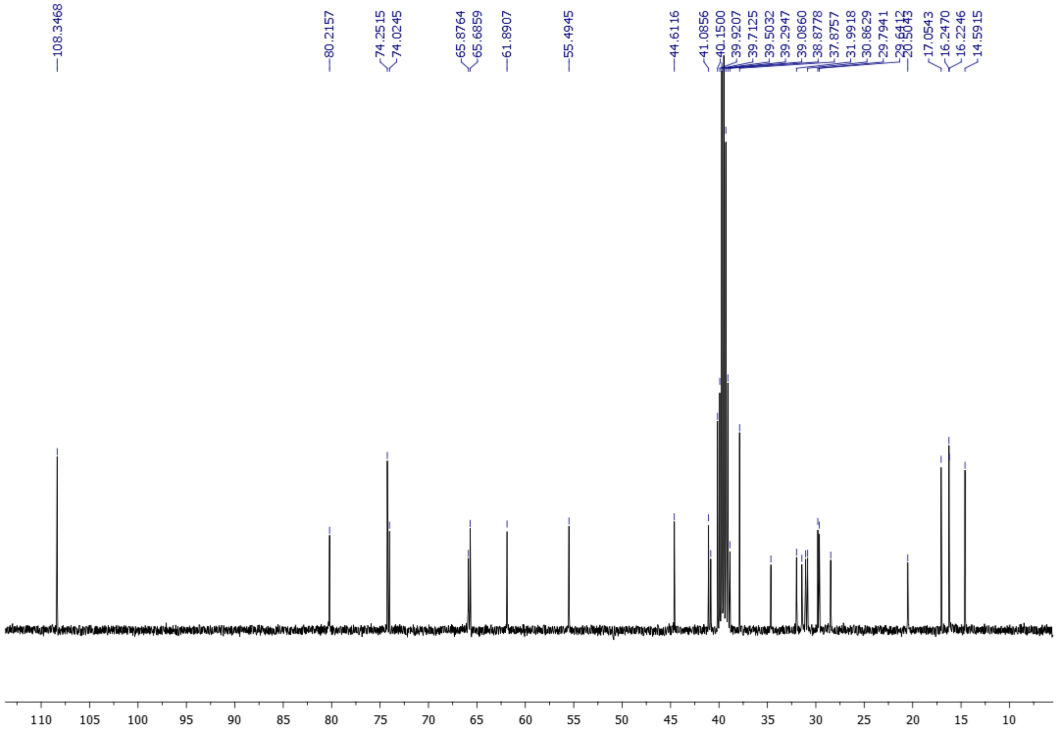

Figure S6: NMR spectra of (25R)-3β,5α-dihydroxy-espirostan-6-ona (**14**)

^{1}H NMR

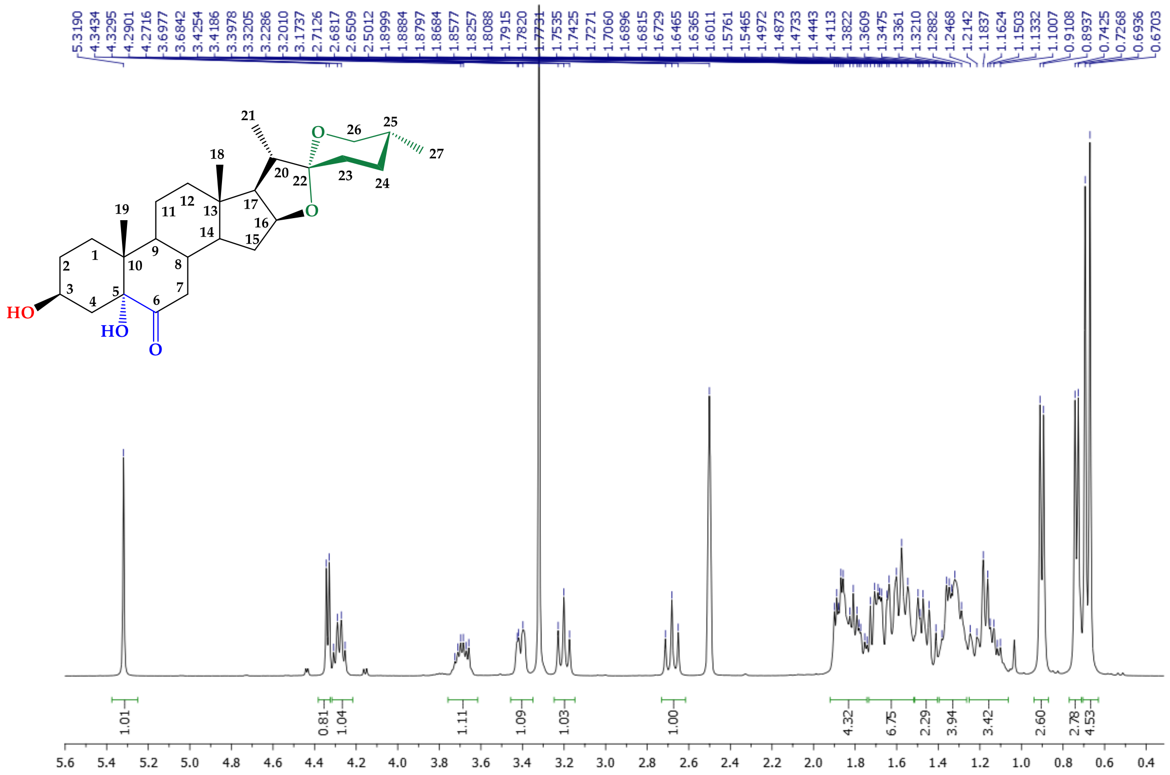

^{13}C-NMR

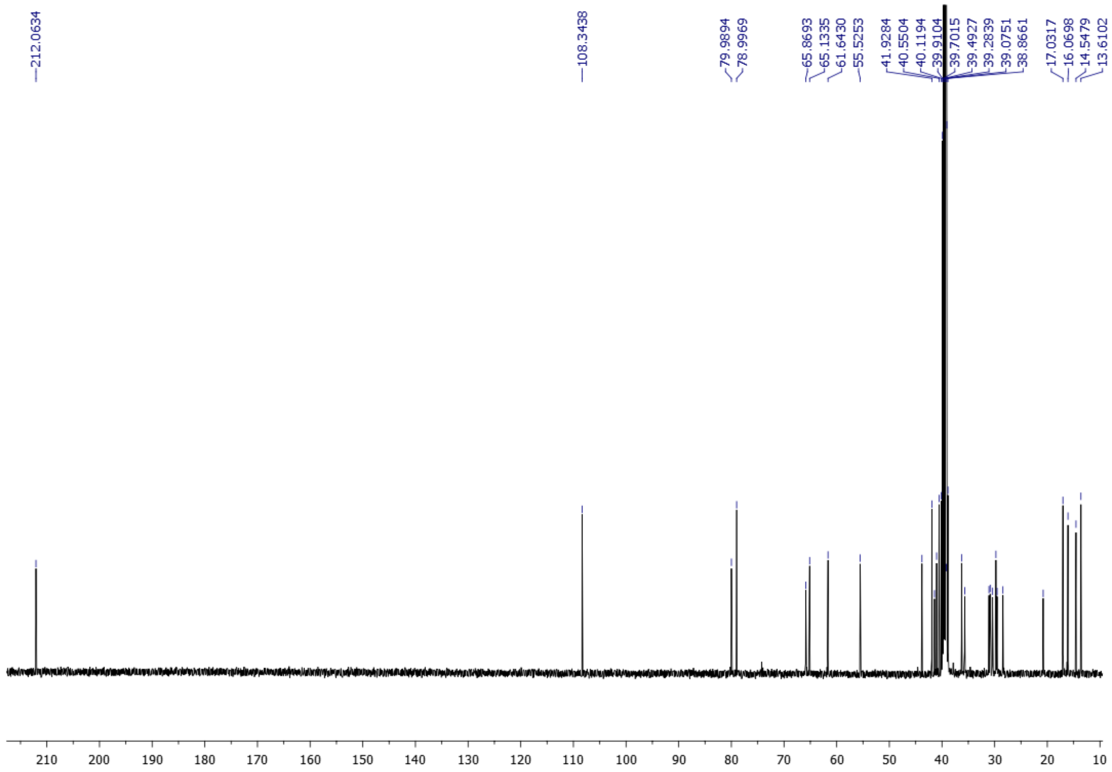

Figure S7: NMR spectra of (25R)- 5α- hydroxy-6-oxo-spirostan-3β-yl phenylcarbamate (**15**)

^{1}H NMR

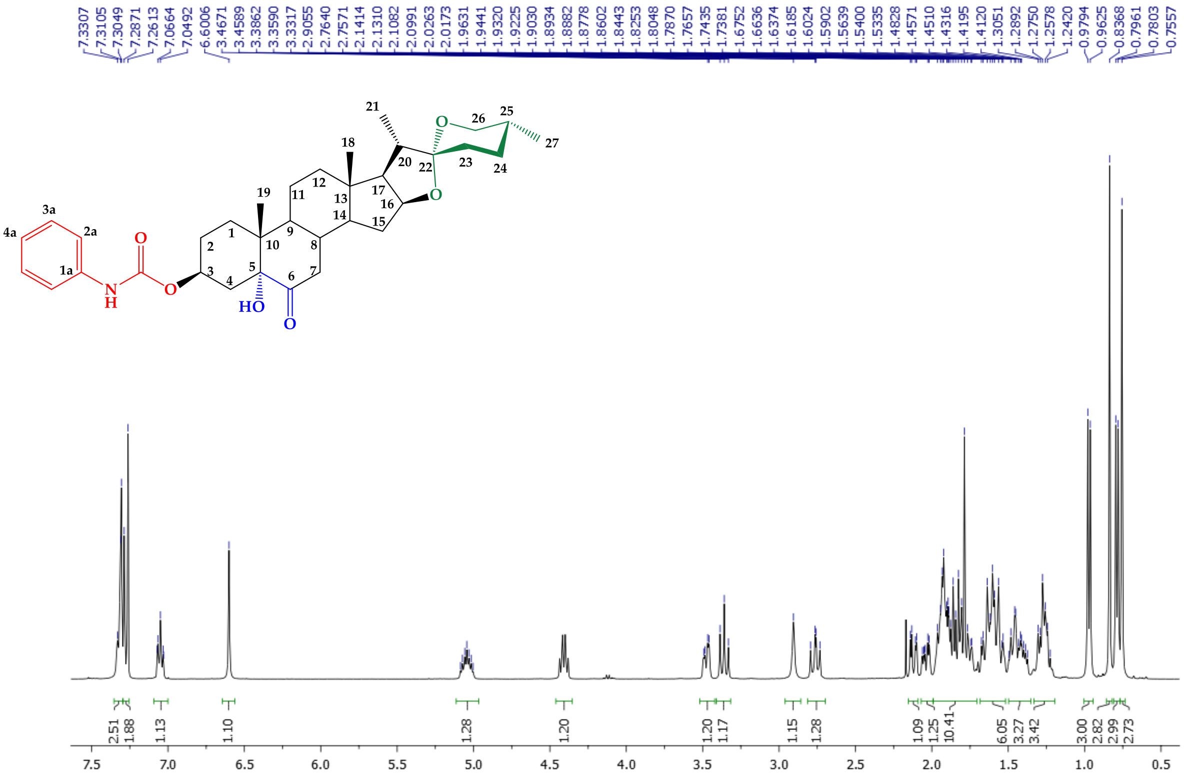

^{13}C-NMR

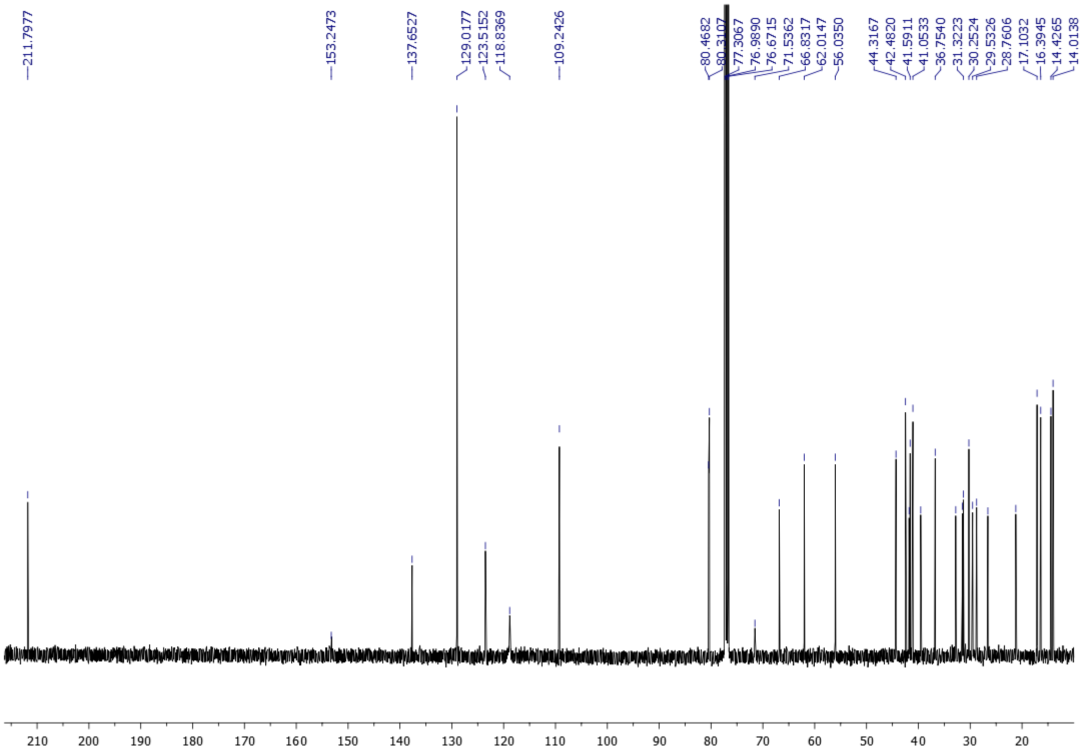

Figure S8: NMR spectra of mixture (25R)-5α,6α-epoxy-spirostan-3β-yl phenylcarbamate (**16a**) and (25R)-5β,6β-epoxy-spirostan-3β-yl phenylcarbamate (**16b**)

^{1}H NMR

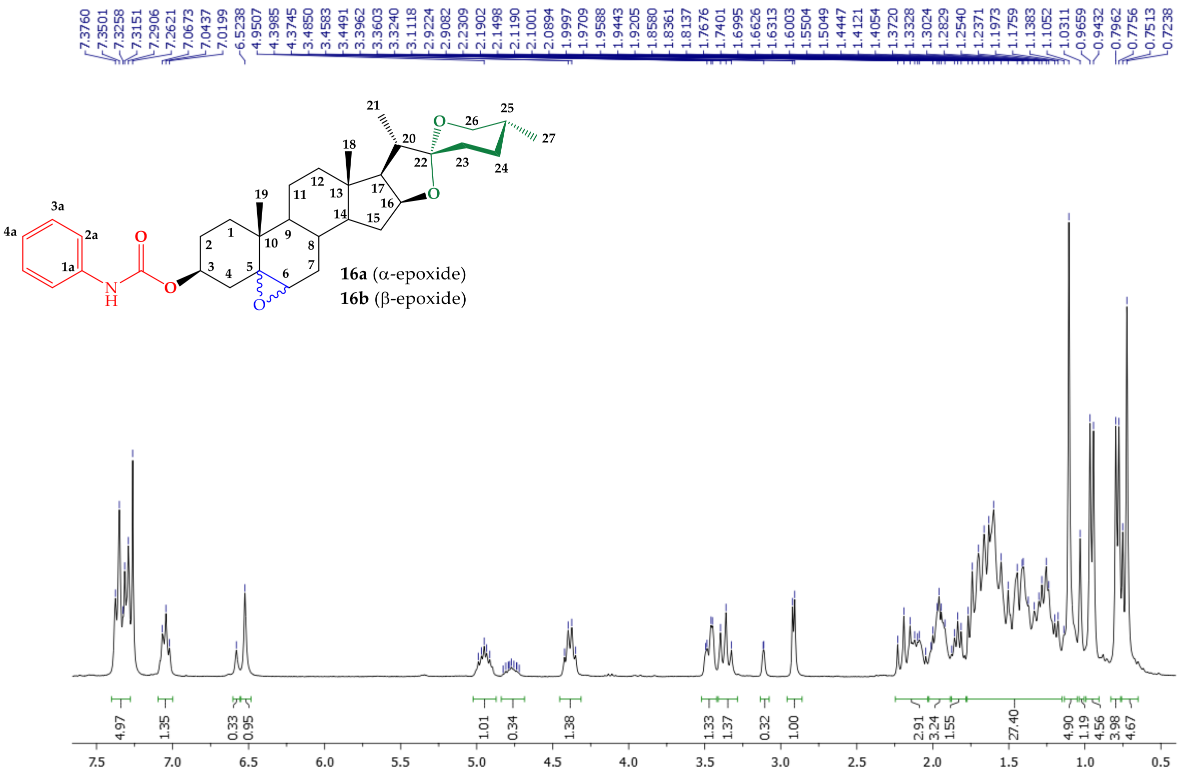

^{13}C-NMR

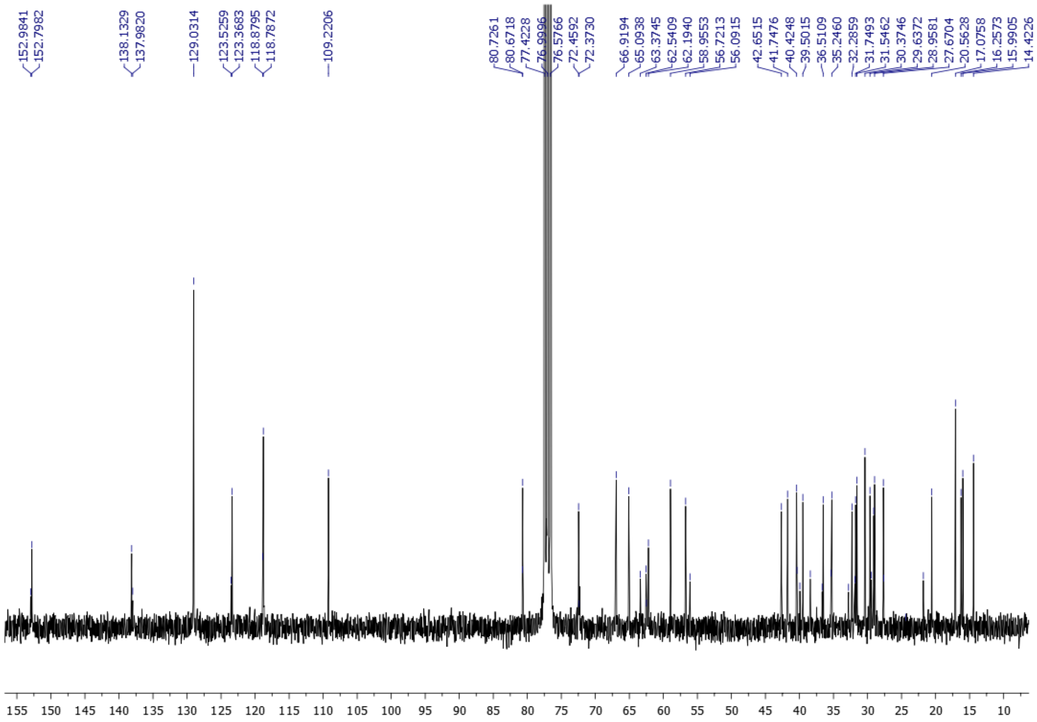

Figure S9: NMR spectra of (25R)-5α-hydroxy-6-hydroxymino-3β-yl phenylcarbamate (**17**)

^{1}H NMR

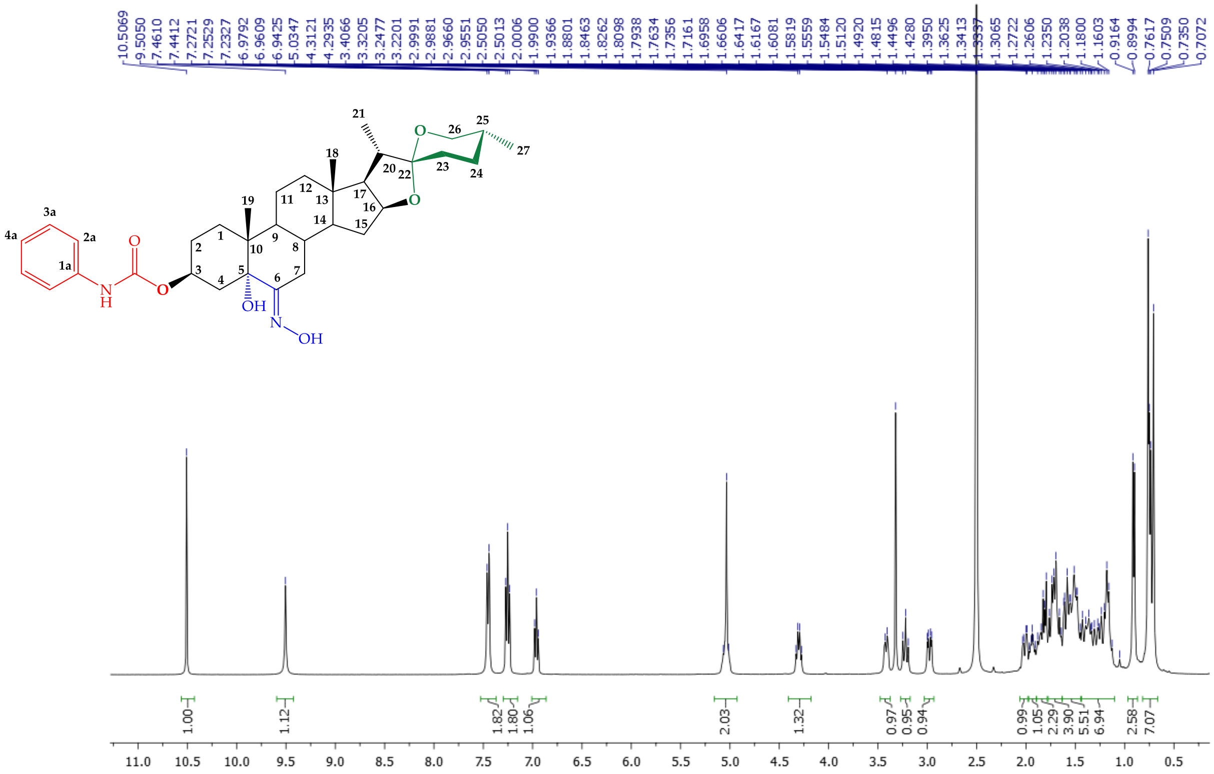

^{13}C-NMR

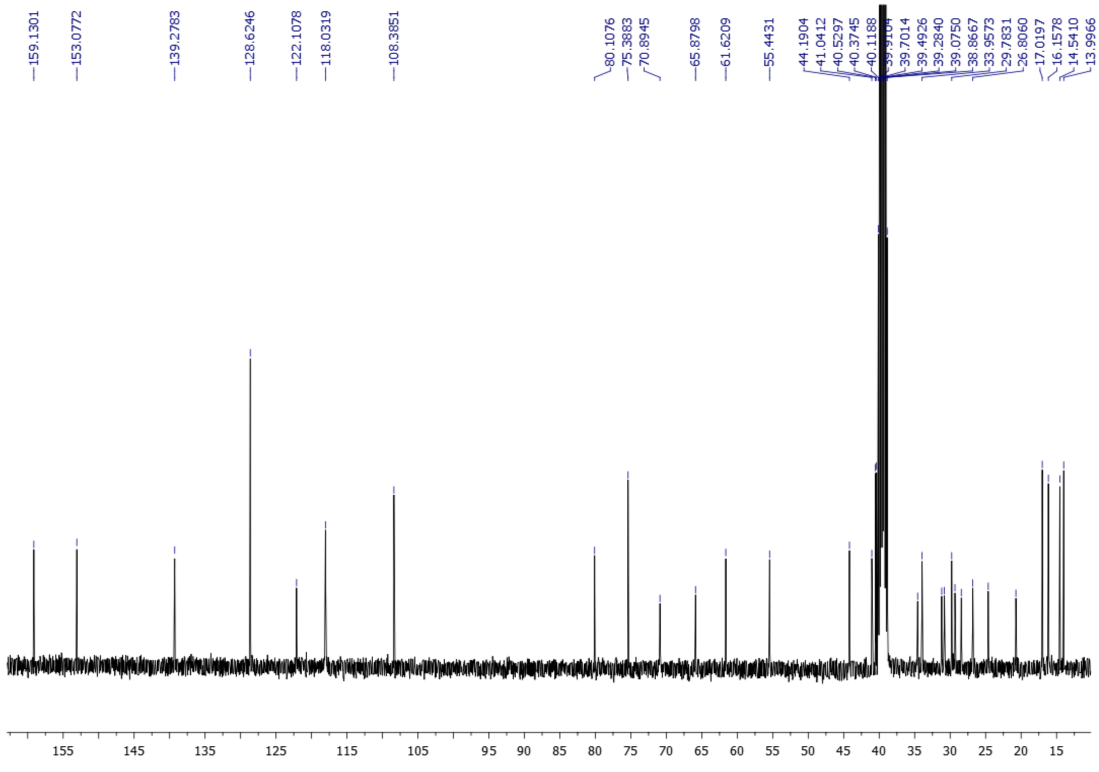

Figure S10: NMR spectra of (25R)-5α,6β-dihydroxy-spirostan-3β-yl phenylcarbamate (**18**)

^{1}H NMR

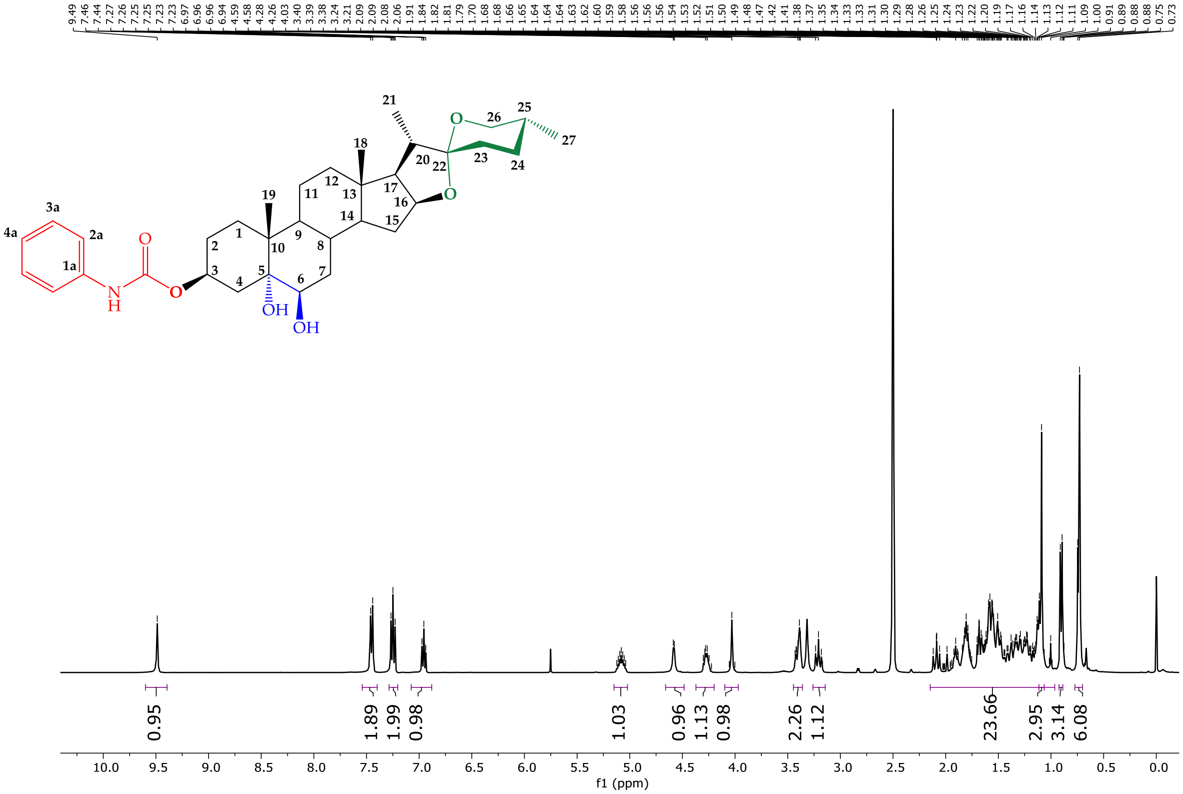

^{13}C-NMR

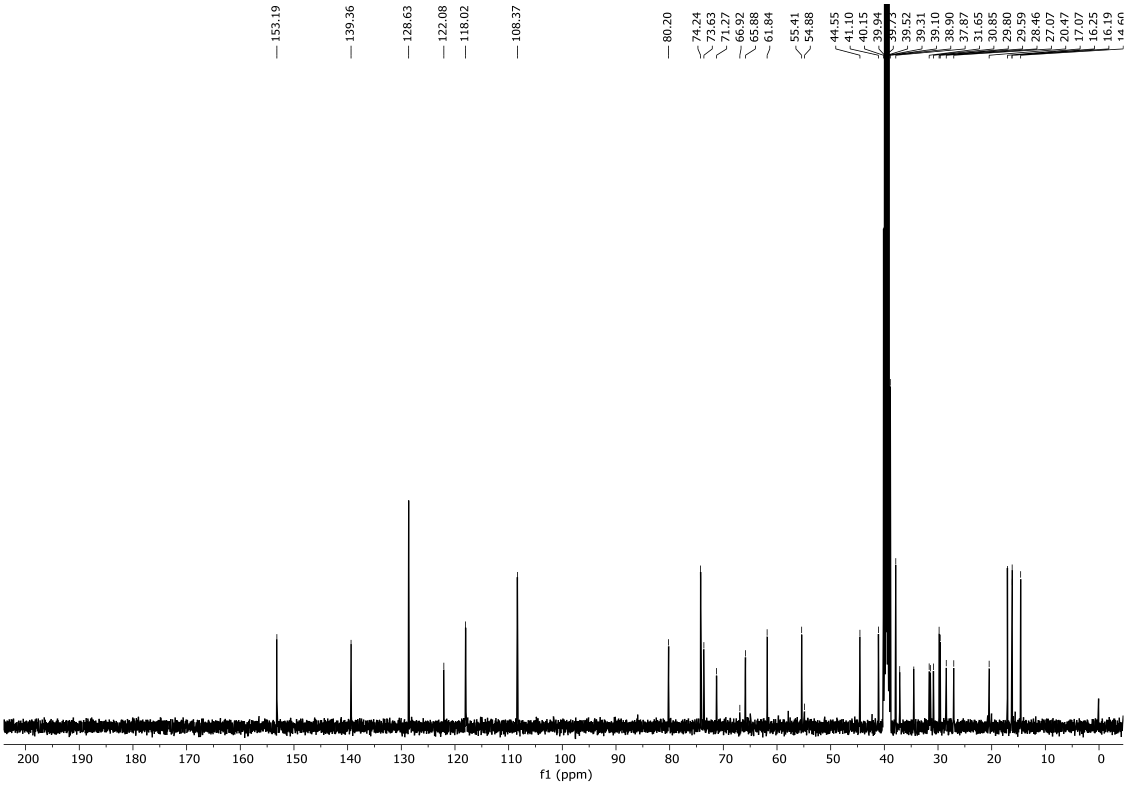

Figure S11: Representations of ligand-BRI1/BAK1 complexes. Crystallographic poses of carbamates steroids **7**, **9**, **11**, **12a**, **12b**, **13**, **14**, **15**, **16a**, **16b**, **17** and **18**. Crystallographic pose of **1** is in black sticks.

Compound **7**

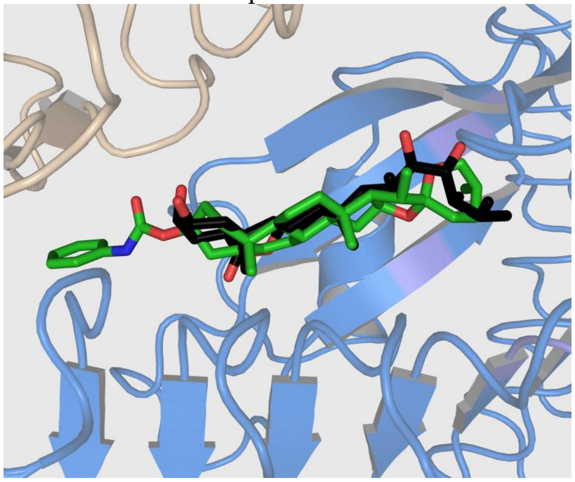

Compound **9** (mode 1):

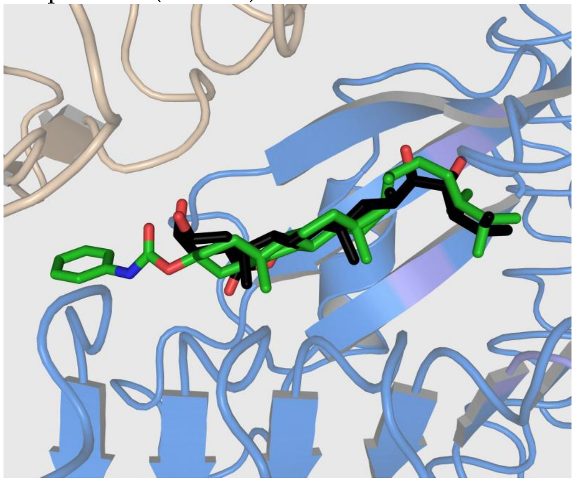

Compound **9** (mode 2):

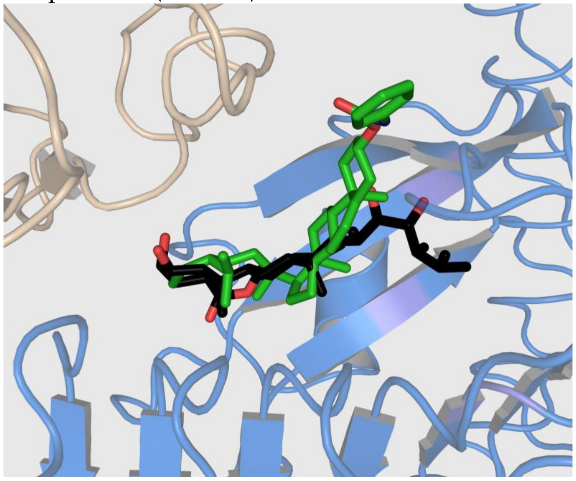

Compound **11** (mode 1):

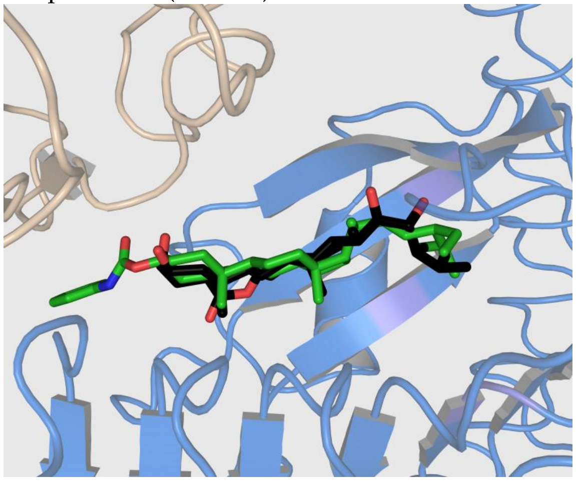

Compound **11** (mode 2):

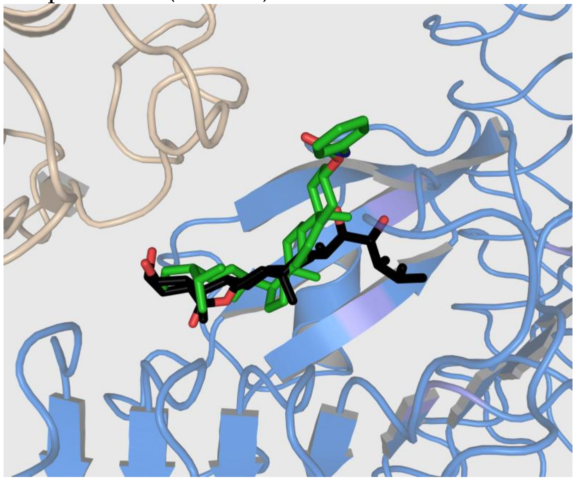

Compound **15**:

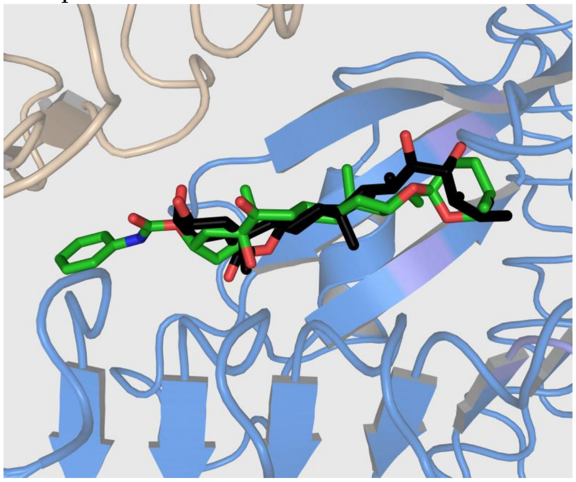

Compound **16a**:

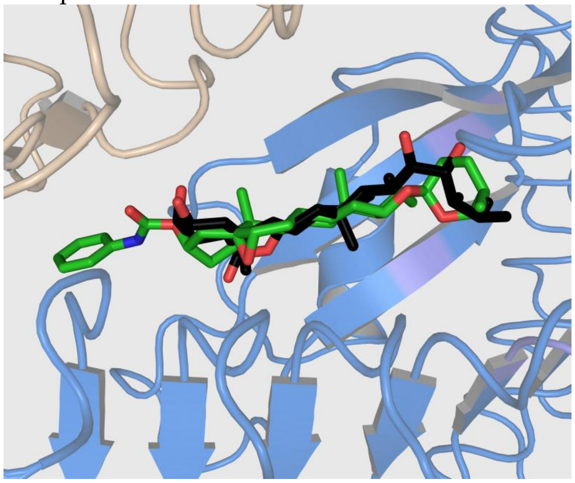

Compound **16b**:

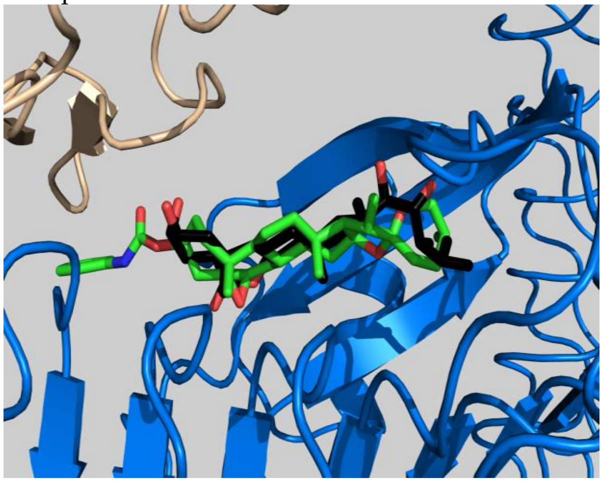

Compound **17** (mode 1):

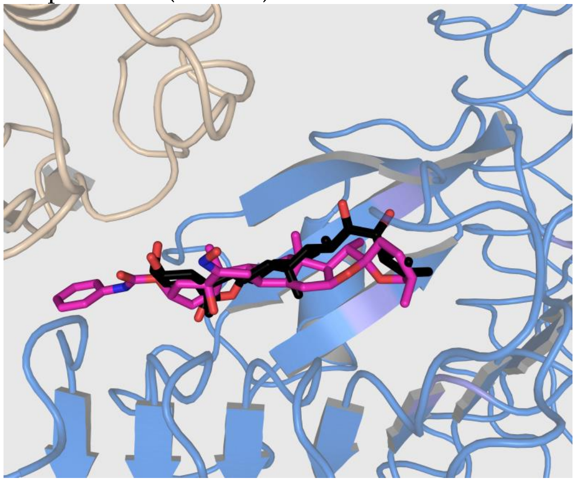

Compound **17** (mode 2):

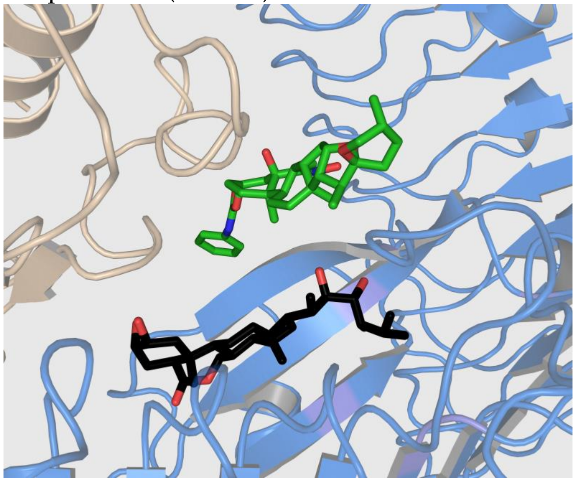

Compound **18** (mode 1):

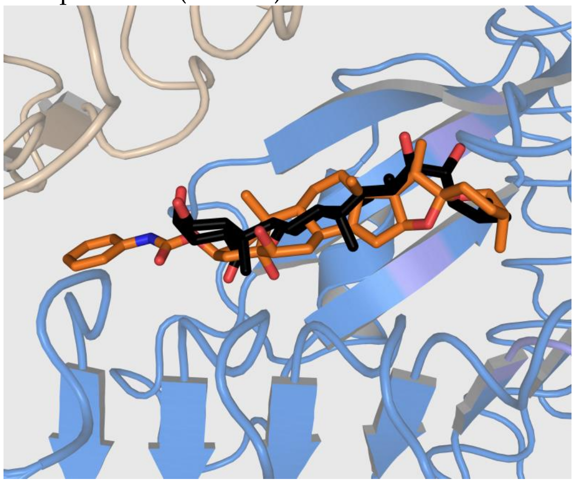

Compound **18** (mode 2):

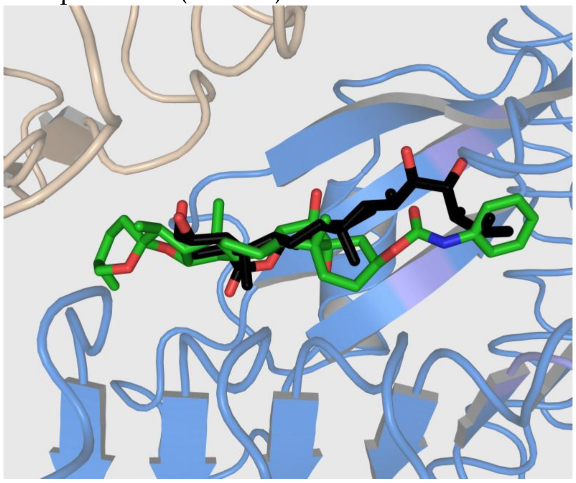

Figure S12: Comparison between the interactions of heterodimer BRI1/BAK1 with **1** and steroidal carbamates ligands

Residues circled in red indicate shared interactions.

Compound **7**-BRI1/BAK1

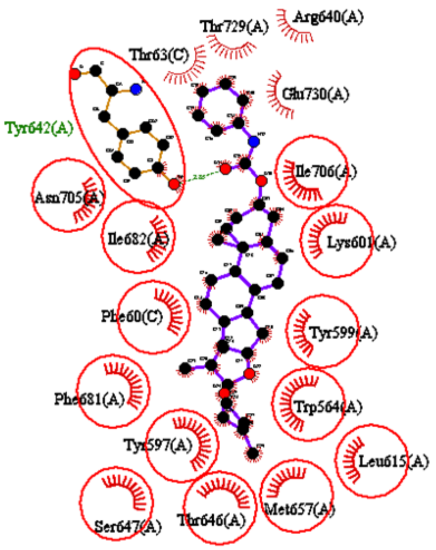

1-BRI1/BAK1

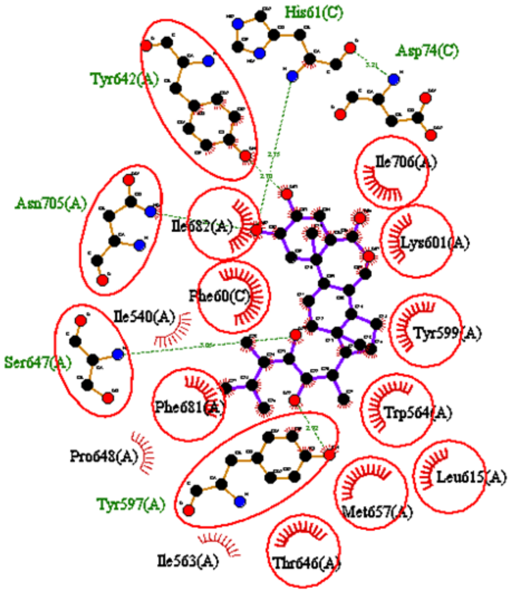

Compound **9**-BRI1/BAK1 (mode 1)

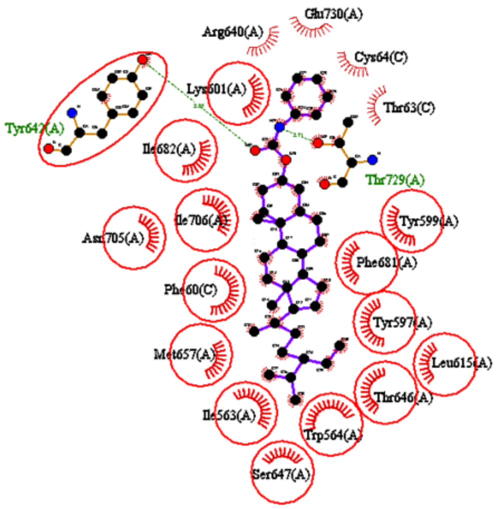

1-BRI1/BAK1

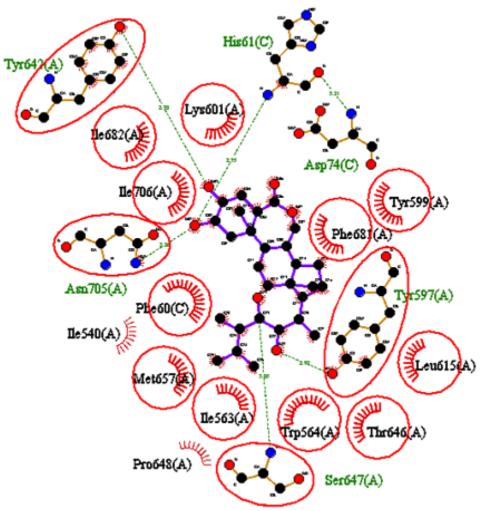

Compound **9**-BRI1/BAK1 (mode 2)

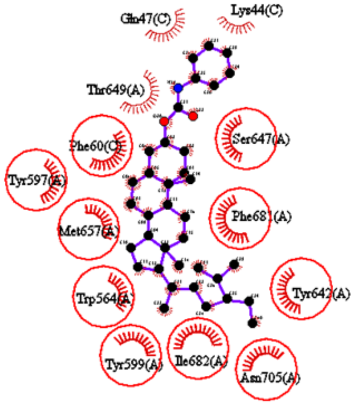

1-BRI1/BAK1

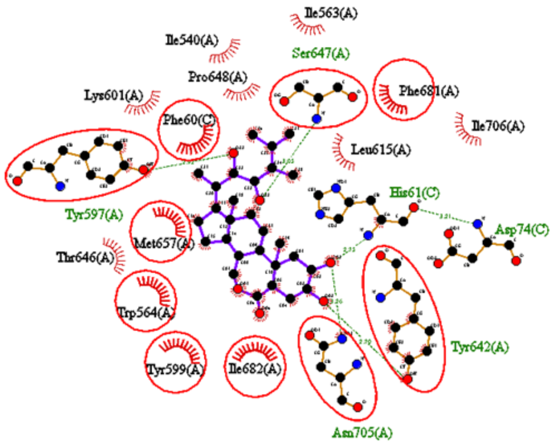

Compound **11**-BRI1/BAK1 (mode 1)

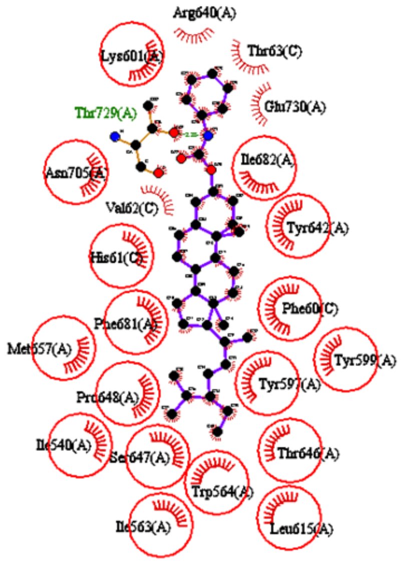

1-BRI1/BAK1

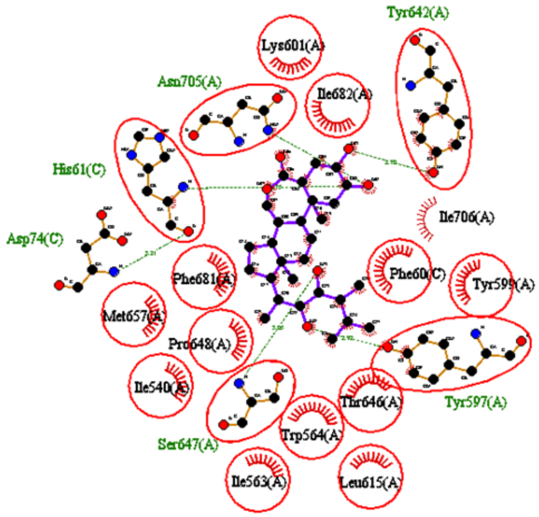

Compound **11**-BRI1/BAK1 (mode 2)

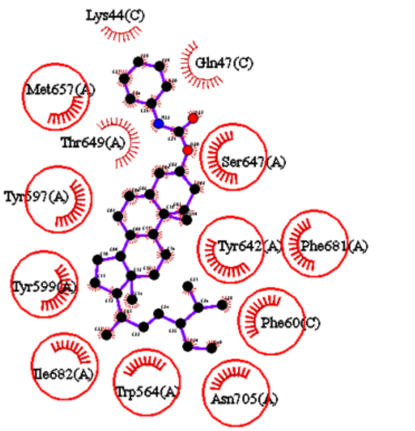

1-BRI1/BAK1

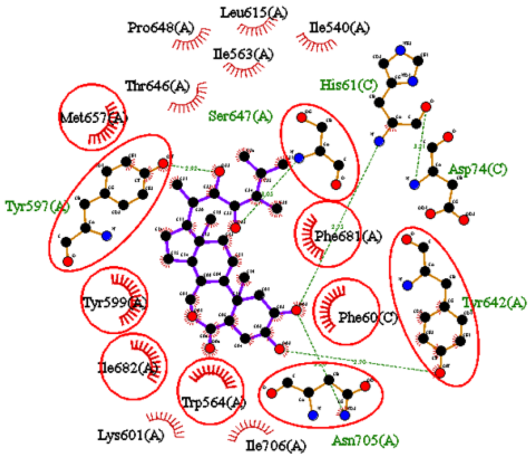

Compound **15**-BRI1/BAK1

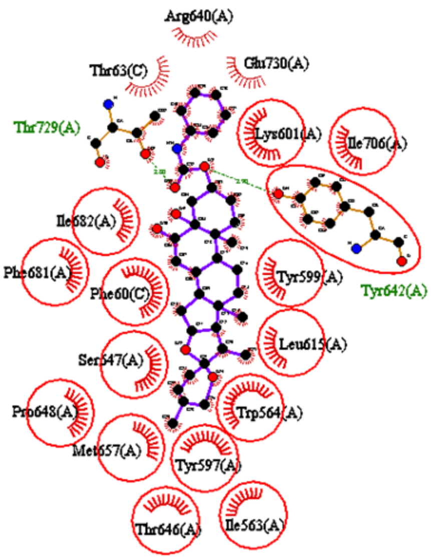

1-BRI1/BAK1

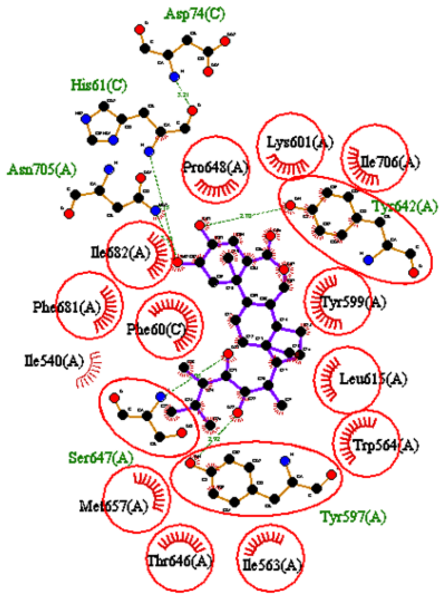

Compound **16a**-BRI1/BAK1

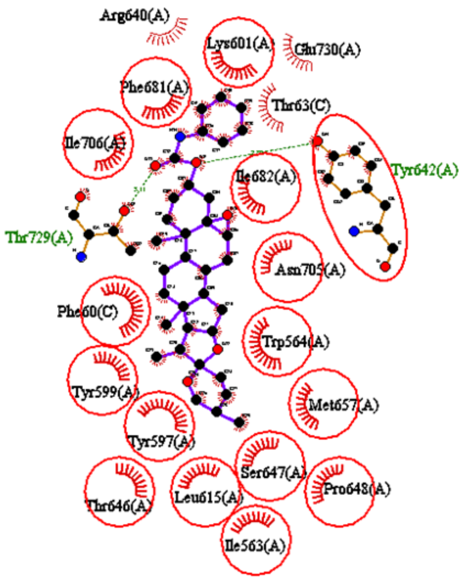

1-BRI1/BAK1

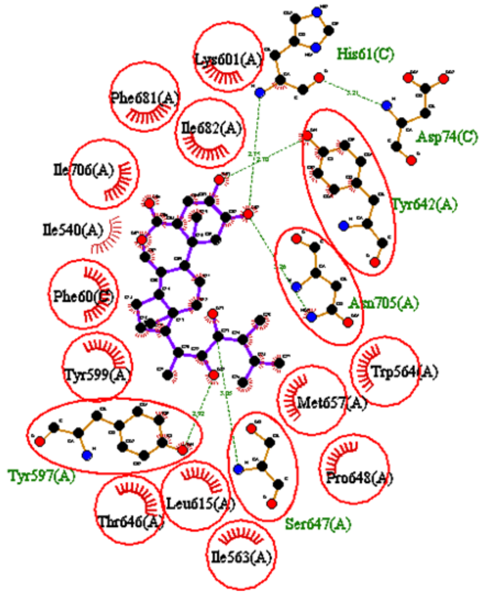

Compound **16b**-BRI1/BAK1

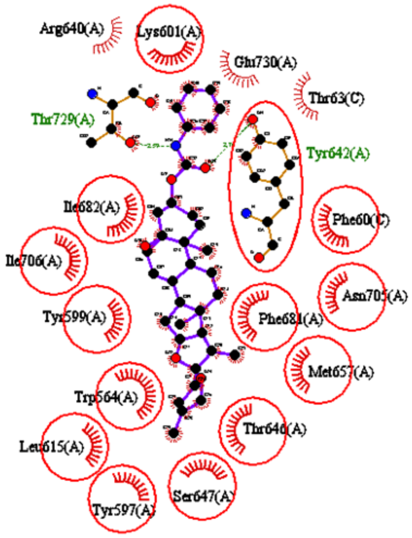

1-BRI1/BAK1

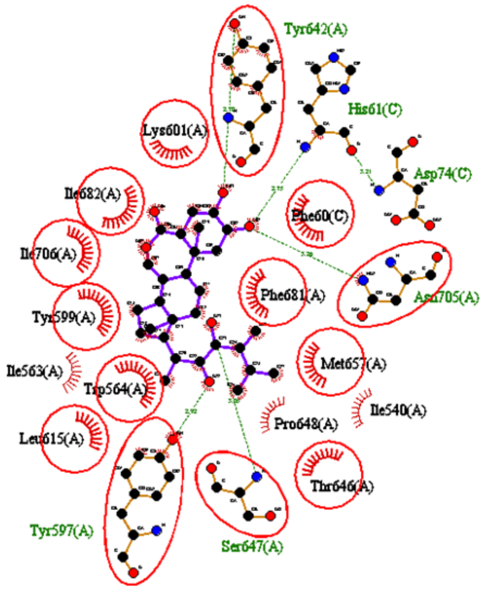

Compound **17**-BRI1/BAK1 (mode 1)

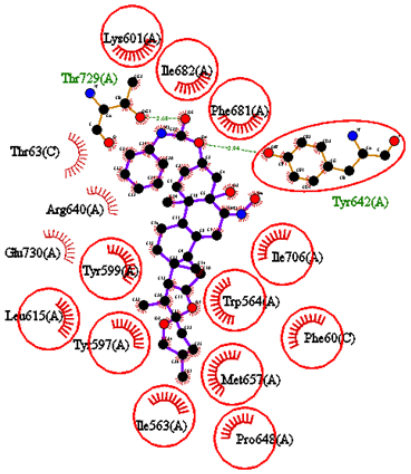

1-BRI1/BAK1

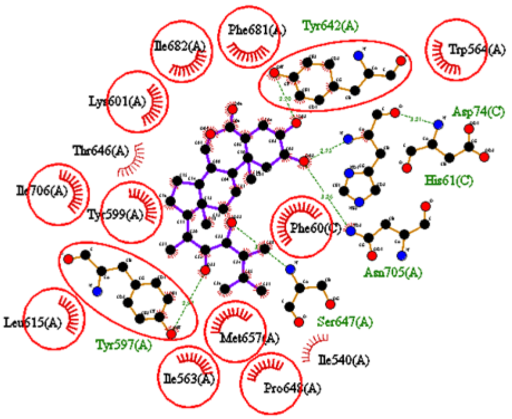

Compound **17**-BRI1/BAK1 (mode 2)

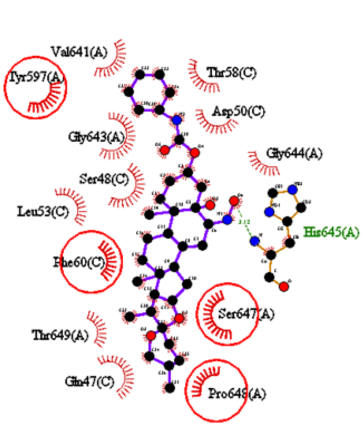

1-BRI1/BAK1

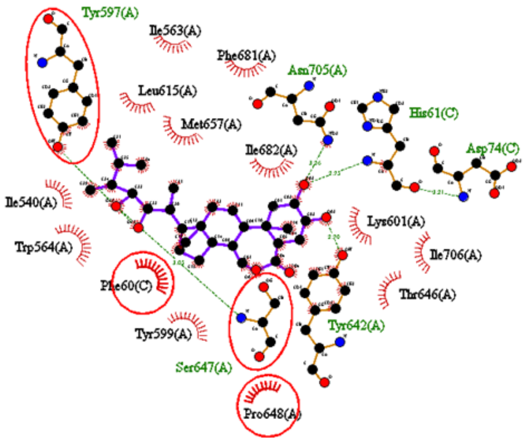

International Journal of *Molecular Sciences*

MDPI

Compound **18**-BRI1/BAK1 (mode 1)

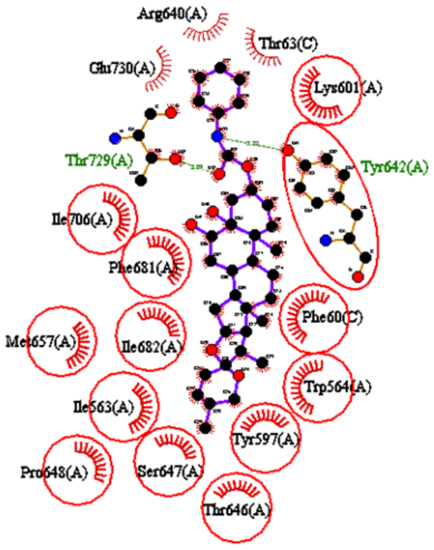

1-BRI1/BAK1

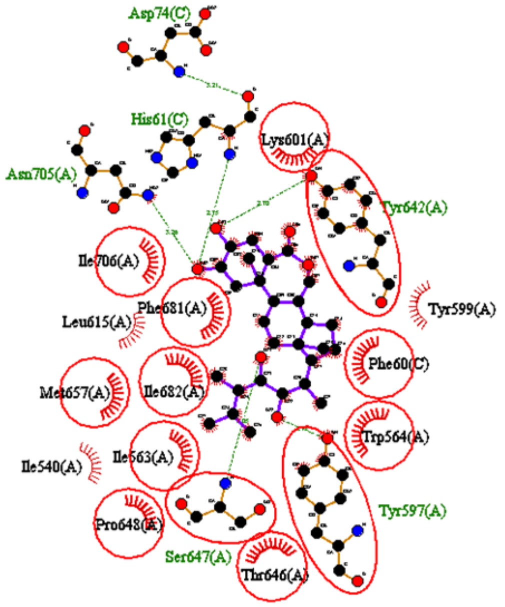

Compound **18**-BRI1/BAK1 (mode 2)

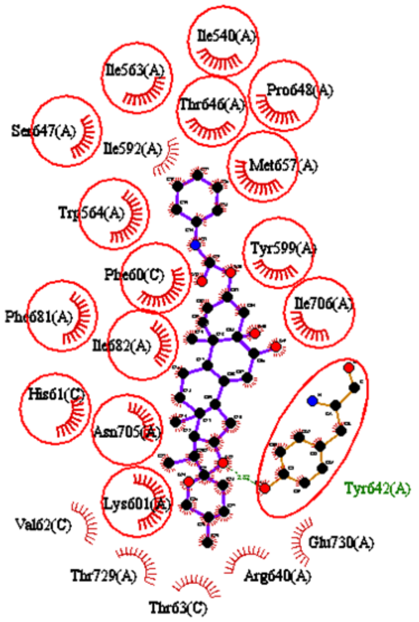

1-BRI1/BAK1

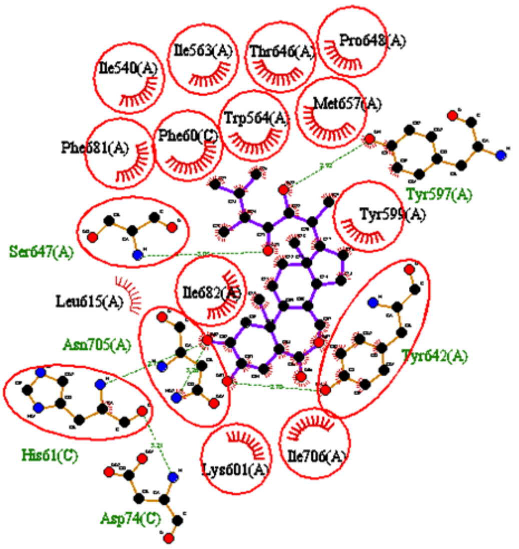

Table S1: Energy (kcal/mol) and conformation clusters of the docked ligands.

Code	Mode 1		Mode 2		Structure
	Number of poses	ΔG_b	Number of poses	ΔG_b	
1	15	-12.6	15	-10.8	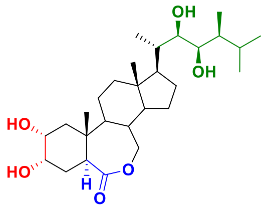
7	15	-12.7	-	-	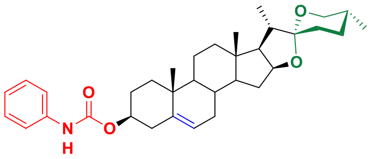
9	15	-11.5	15	-10.9	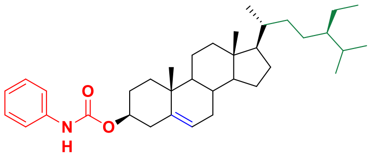
11	14	-11.6	14	-11.5	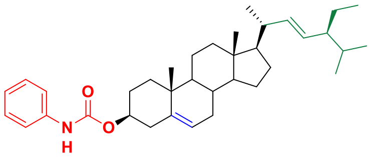
15	15	-13.1	-	-	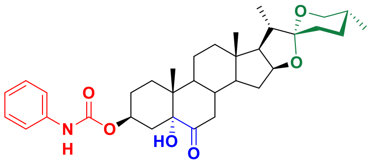
16a	15	-12.9	-	-	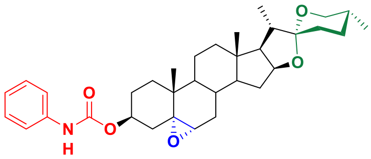
16b	15	-12.1	-	-	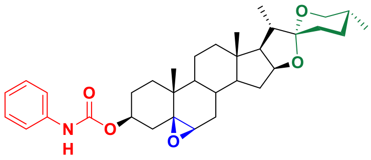

17	15	-9.1	12	-7.1	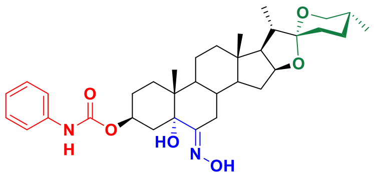

© 2017 by the authors. Submitted for possible open access publication under the terms and conditions of the Creative Commons Attribution (CC BY) license (http://creativecommons.org/licenses/by/4.0/).
